# Impact of exposure to malaria and nutritional status on responses to the experimental malaria vaccine ChAd63 MVA ME-TRAP in 5-17 month-old children in Burkina Faso

**DOI:** 10.3389/fimmu.2022.1058227

**Published:** 2022-12-02

**Authors:** Richard Morter, Alfred B. Tiono, Issa Nébié, Oliver Hague, Alphonse Ouedraogo, Amidou Diarra, Nicola K. Viebig, Adrian V. S. Hill, Katie J. Ewer, Sodiomon B. Sirima

**Affiliations:** ^1^ Nuffield Department of Clinical Medicine, The Jenner Institute, University of Oxford, Oxford, United Kingdom; ^2^ Centre National de Recherche et de Formation sur le Paludisme, Ouagadougou, Burkina Faso; ^3^ Groupe de Recherche Action en Santé (GRAS), Ouagadougou, Burkina Faso; ^4^ European Vaccine Initiative, UniversitätsKlinikum Heidelberg, Heidelberg, Germany

**Keywords:** malaria, vaccine, viral vector, ME-TRAP, Burkina Faso, immunosuppression, nutrition

## Abstract

The experimental malaria vaccine ChAd63 MVA ME-TRAP previously showed protective efficacy against *Plasmodium falciparum* infection in Phase IIa sporozoite challenge studies in adults in the United Kingdom and in a Phase IIb field efficacy trial in Kenyan adults. However, it failed to demonstrate efficacy in a phase IIb trial in 5-17 month-old children in an area of high malaria transmission in Burkina Faso. This secondary analysis investigated whether exposure to malaria or nutritional status might be associated with reduced responses to vaccination in this cohort. Parasite blood smears and anti-AMA-1 IgG titres were used to assess history of exposure to malaria and weight-for-length Z scores were calculated to assess nutritional status. Differences in vaccine-specific anti-TRAP IgG titre and *ex vivo* IFNγ ELISpot response were measured between groups. In total, *n* = 336 volunteers randomised to receive the experimental vaccine regimen were included in this analysis. A positive smear microscopy result was associated with reduced anti-TRAP IgG titre (geometric mean titre: 2775 (uninfected) vs 1968 (infected), *p* = 0.025), whilst anti-AMA-1 IgG titres were weakly negatively correlated with reduced *ex vivo* IFNγ ELISpot response (r = -0.18, *p* = 0.008). Nutritional status was not associated with either humoral or cellular immunogenicity. Vaccine efficacy was also measured separately for vaccinees with positive and negative blood smears. Although not significant in either group compared to controls, vaccine efficacy measured by Cox hazard ratio was higher in uninfected compared to infected individuals (19.8% [*p* = 0.50] vs 3.3% [*p* = 0.69]). Overall, this data suggests exposure to malaria may be associated with impaired vaccine immunogenicity. This may have consequences for the testing and eventual deployment of various vaccines, in areas with high endemicity for malaria.

**Trial Registration:**

Pactr.org, identifier PACTR201208000404131; ClinicalTrials.gov, identifier NCT01635647.

## 1 Introduction

Malaria caused by the apicomplexan parasite *Plasmodium* spp. is a leading cause of child mortality worldwide. 627 000 deaths occurred globally from malaria in 2020 alone (1). There were 14 million additional cases compared to 2019, driven partly by disruptions to control efforts resulting from the COVID-19 pandemic. However, even before this, progress in reducing the burden of malaria had stagnated. This highlights the urgent need for additional control measures, such as vaccines (2).

On average 95% of malaria cases occur in sub-Saharan Africa, of which 80% of deaths occur in children below 5 years of age (1). The majority of cases in the region are caused by *P. falciparum.* Following pilot implementation in three African countries, the first vaccine developed against *P. falciparum* malaria, RTS,S/AS01, was recommended by the WHO in October 2021 for wider use in children living in regions with moderate to high transmission (3). Despite this, the vaccine has been shown to provide only partial and relatively short-lived immunity (4–8). There is a need to continue to develop malaria vaccines which might afford greater protection and durability.

Another candidate vaccine regimen; ChAd63 ME-TRAP and MVA ME-TRAP is based on non-replicating viral vector technology (ChAd63 and MVA) expressing the pre-erythrocytic stage antigenic insert ME-TRAP. This vaccine was tested in a Phase IIb clinical trial in 5-17 month old infants in Banfora, Burkina Faso, an area of high endemicity for *P. falciparum*. The vaccine was shown to be safe, however no significant protective efficacy against clinical malaria was observed (9). This was despite the vaccine previously showing 67% efficacy against PCR-determined malaria in Kenyan adults (10). CD8+ T cell responses have been shown to correlate with vaccine efficacy (11) and T cell responses measured by *ex vivo* IFNγ ELISpot assay were substantially lower in this cohort than previously observed in UK, Gambian and Kenyan adults or Gambian infants. Conversely, antibody titres were significantly higher than previously seen in adults in the UK or Africa.

It has long been thought that malaria infection has broad immunosuppressive effects on the host. Some evidence exists showing malaria-mediated suppression of vaccine responses (12), including other T cell-inducing ME-TRAP based malaria vaccines (13). However, this is not often investigated in clinical trials of malaria vaccines in endemic settings. The mechanisms by which immunosuppression is thought to occur are complex and largely not understood (14). As well as malaria infection, another important factor which may cause immunosuppression in this cohort is malnutrition (15). It is important to understand the mechanisms by which vaccine immunogenicity and efficacy is reduced in groups which would most benefit from their use to devise strategies to mitigate these effects.

This study is a follow-up to the data previously published reporting on the safety, efficacy and immunogenicity of the candidate malaria vaccine ChAd63 MVA ME-TRAP in a Phase IIb clinical trial in 5-17 month-old infants and children in Banfora, Burkina Faso (9). Here, we investigate whether malnutrition or exposure to malaria infection correlates with vaccine immunogenicity or efficacy. Investigating what factors might be associated with reduced vaccine responses is important for exploring potential mechanisms of immunosuppression, to inform the design of next-generation vaccines.

## 2 Materials and methods

### 2.1 Clinical trial study design

Full details on the design and conduct of the clinical trial from which samples were derived for this analysis can be found in the primary clinical trial publication (9) and the clinical trial protocol included in the appendix. Briefly, a Phase IIb, double blind, randomised control trial of a candidate malaria vaccine, ChAd63 MVA ME-TRAP was conducted in Banfora, Burkina Faso, West Africa. This is an area of high endemicity for *P. falciparum* malaria with peak transmission during the rainy season (between June to October).

The clinical trial protocol and associated documents were reviewed and approved by Centre National de Recherche et de Formation sur le Paludisme (CNRFP) institutional bioethics committee (approval reference N° 2012/04/MS/SG/CNRFP/CIB), the Ministry of Health Ethical Committee for Biomedical Research (Burkina Faso) (approval reference N° 2012-6-37) and Oxford Tropical Research Ethics Committee (OXTREC) (approval reference N° 41–12). Regulatory approval was given in Burkina Faso by the national regulatory authority (Comité Technique pour les Essais Cliniques, CTEC). Informed consent was given prior to enrolment by the study participants’ parents or legally acceptable representatives. Ethical review and informed consent included approval for the re-analysis of samples for exploratory immunology including transcriptomic and genetic analysis. The trial was conducted according to the principles of the Declaration of Helsinki and International Conference on Harmonization (ICH) Good Clinical Practice (GCP) guidelines. An independent data safety monitoring board (DSMB) and local safety monitors provided safety oversight and GCP compliance was independently monitored by an external organisation (Appledown Clinical Research Ltd., Buckinghamshire, UK). The study was registered at ClinicalTrials.gov [NCT01635647] and Pactr.org [PACTR201208000404131].

Following community sensitisation, parents and participant infants aged between 5-17 months were invited to screening to assess eligibility and to collect basic demographic and anthropometric data including height and weight. Healthy participants meeting the inclusion and exclusion criteria (appended in the study protocol) were then enrolled into the trial, conducted between December 2012 and March 2014. Participants were not screened for malaria exposure prior to enrolment. In total, 700 participants were randomised 1:1 to receive either the candidate malaria vaccine regimen; 5 x 10^10^ vp ChAd63 ME-TRAP followed by 1 x 10^8^ pfu MVA ME-TRAP, or the control vaccine; Imovax Rabies Vaccine (Sanofi Pasteur SA, France). For both trial arms, two vaccine doses were given at an interval of 8 weeks.

Volunteers were monitored for solicited and unsolicited adverse events by study clinicians or trained field workers and were followed-up for malaria infection for 6 months following the last vaccination. Parents were advised to contact the study team if their child became unwell at any point during follow-up, in order to access medical treatment. Any symptoms compatible with malaria (including but not limited to; axillary temperature ≥ 37.5°C, history of fever within the last 24 hours, loss of appetite, malaise, vomiting and diarrhoea) were investigated with duplicate blood films and a malaria rapid diagnostic test. Positive diagnoses were promptly treated according to national guidelines and data recorded for analysis of vaccine efficacy. Only blood film results were used for efficacy analysis, as per the study protocol.

The analysis presented here was performed after unblinding and therefore does not include data from volunteers in the rabies control group.

### 2.2 Laboratory investigations

#### 2.2.1 Assessment of parasitaemia

Blood films were obtained by capillary sampling at screening, vaccination and pre-defined timepoints during follow-up, as well as in infants presenting acutely unwell with symptoms compatible with malaria infection. Thick and thin blood films were stained by Giemsa staining using standard methods and read under 100 x bright field microscopy by trained laboratory technicians for the assessment, speciation, and quantification of parasitaemia. The count was made by species (*P*. *falciparum*, *P*. *malariae*, or *P*. *ovale*), and counts for *P*. *falciparum* were made for both sexual and asexual parasites. The parasite presence and density were determined independently by two readers for the same slide; if readings were judged to be discordant, a third independent read was organised. The parasite density (parasites/μl) was calculated as the geometric mean of the two positive readings (two geometrically closest readings in the case of three positive reads).

#### 2.2.2 Blood sampling for exploratory immunology

Venous blood was collected at pre-defined timepoints from all volunteers who received the experimental vaccine regimen as well as a subset of *n* = 50 volunteers who received the rabies control vaccine, in order to preserve blinding. Data from rabies control group volunteers has not been included here, as this analysis was performed after unblinding. Between 5-8 ml of blood was collected into lithium-heparin collection bottles (Becton Dickinson, UK) for the isolation of peripheral blood mononucleocytes (PBMCs) and plasma by standard methods using density gradient centrifugation with Lymphoprep (Axis-Shield Diagnostics Ltd., UK). These methods have been described previously (16)

#### 2.2.3 *Ex-vivo* IFNγ ELISpot

*Ex vivo* IFNγ ELISpot assays were performed on fresh PBMC to assess the magnitude and kinetics of the T cell response as previously described (9, 11). Sterile Multiscreen IP ELISpot plates (Millipore, USA) were coated with human IFNγ SA-ALP antibody kits (Mabtech, Sweden). Cells were plated in duplicate at 2.5 x 10^5^ cells/well and stimulated with synthesised peptide pools of 20mer peptides overlapping by 10 amino acids, with up to 10 peptides per pool at a concentration of 10 μg/ml (NEOpeptide, USA). Peptides spanned the length of the ME-TRAP antigenic insert of both the T9/96 and 3D7 strains of *P. falciparum*. Peptide sequences have been published previously (11). Negative (DMSO (Sigma-Aldrich, USA) only) and positive (0.02 μg/ml staphylococcal enterotoxin B with 10 μg/ml phytohaemagglutinin-L (Sigma-Aldrich)) controls were also included for each sample. Cells were suspended in RPMI media (Sigma-Aldrich) with 10% heat-inactivated foetal calf serum (Labtech International, UK). Stimulations were left to occur for 18-20 hours before development with BCIP NBT-plus substrate (Moss Inc., USA). Plates were counted using an ELISpot counter (Autoimmun Diagnostika (AID), Germany) with standardised settings and counts only adjusted to remove artifacts. Results are expressed as the mean of the duplicate IFNγ spot-forming cells (SFC) per million PBMC, subtracted from the background responses in negative control wells and summed across all ME-TRAP (T9/96) peptide pools. Any samples with counts >80 SFC per million PBMC in the negative control well were excluded from analysis.

#### 2.2.4 TRAP- and AMA-1-specific total IgG ELISA

Total anti-TRAP and anti-AMA-1 IgG titres were measured separately using standardised sandwich ELISAs as previously described (9, 11, 17). Nunc-Immuno MaxiSorp plates (ThermoFisher, USA) were coated with recombinant TRAP (1 μg/ml) or AMA-1 protein (3D7 strain) (2 μg/ml) in PBS and blocked for 1 hour with Casein block solution (Pierce, UK). Test samples were plated in triplicate and standard samples in duplicate on each plate. 50 μl of sample was plated per well. Samples were incubated for 2 hours before addition of goat anti-human IgG (γ chain) conjugated to alkaline phosphatase (Sigma-Aldrich) for 1 hour. Plates were developed with p-nitrophenylphosphate at 1 mg/ml in diethanolamine buffer (Pierce) and read at 405 nm on an Elx800 microplate reader (BioTek, USA). Test samples were diluted to a minimum of 1:100 for the TRAP assay and 1:300 for the AMA-1 assay. For the TRAP assay, a standard curve was derived using sera from *n* = 30 high-responding vaccinated volunteers, initially diluted to 1:100 and serially diluted 1:3 thereafter. For the AMA-1 standard curve, sera from *n* = 25 hyperimmune individuals from Kilifi, Kenya was initially diluted 1:1000 and serially diluted 1:2 thereafter. Each dilution on the standard curve was assigned an arbitrary value in ELISA units (EU). Standard curves were modelled using five parameter logistic curve fitting and test plasma endpoint titres were inferred from the *x* axis intercept values of dilutions which lay within the linear range of the curve. Seropositive cut-off values for each assay were established using the mean plus three standard deviations of the EU readings of 42 unvaccinated and malaria-naïve UK donor plasma samples. For the TRAP assay, this cut-off was 88 EU and for AMA-1, this was 14 EU.

### 2.3 Statistical analysis

For exploratory immunology analysis, data were assessed by D’Agostino-Pearson normality testing and determined to be non-normally distributed. Non-parametric tests were applied using Prism (version 9, GraphPad Software Inc., USA). Weight-for-length Z scores were calculated using the WHO Child Growth Standards with the igrowup macro (18) in STATA software (version 16, StataCorp LLC, USA).

For calculation of efficacy, the incidence of first or only episodes of *P. falciparum* malaria (episodes/person years at risk) were calculated for each group. The distribution of the survival time was compared with the Wilcoxon test (if efficacy appeared to vary with time) or the Log-rank test (if it did not). Vaccine efficacy was assessed using Cox regression models for the first episode. Vaccine efficacy is defined as 1-R where R is the hazard ratio of the malaria vaccine group (infected or uninfected) versus the rabies control group (with 95% CI). A primary case of malaria episode was defined as fever (axillary temperature ≥ 37.5°C) with *P. falciparum* count > 5,000 trophozoites/µL of blood. The secondary case definition is defined as fever (axillary temperature ≥ 37.5°C) with *Plasmodium falciparum* count > 0 trophozoites/µL of blood. Analyses were performed using STATA software (version 15) and GraphPad Prism (version 8 and 9).

A *p* value of 0.05 was used as the threshold of statistical significance.

## 3 Results

### 3.1 Study population characteristics

Phase IIb safety, immunogenicity and efficacy results were previously published (9). Briefly, 700 5-17 month-old infants and children in Banfora, Burkina Faso, were randomised to receive either ChAd63 ME-TRAP followed by MVA ME-TRAP 8 weeks later, or a control rabies vaccine. Vaccination was found to be safe and moderately immunogenic, with T cell responses lower than seen in previous trials [median ELISpot; 326 SFC/10^6^ PBMC (95% CI: 290–387)] at one week post-boost (day 63). Antibody responses were similar to previous trials [median titre; 3467 EU (95% CI: 2849–4168)], however, higher titres were observed in females compared to males [*p* = <0.004]. No significant efficacy between vaccinees and controls against uncomplicated malaria was observed [vaccine efficacy; 13.8%; 95%CI: -42.4 to 47.9]. Efficacy against severe malaria was found to be 19.4% [95%CI: -58.9 to 59.1; *p* = 0.53] and -4.7% [-114.0 to 48.8, *p* = 0.9] in unadjusted and adjusted cohorts respectively (per protocol analysis by Cox regression).

In total, 351 participants were assigned to the experimental malaria vaccine group. From these, 336 received both doses of vaccine per-protocol and are included in the analysis presented here. Participants were sampled at day 0 (baseline) and day 63 (one week post-boost) for collection of blood plasma and PBMCs for immunological analysis. Participants were also bled for smear microscopy at day 0 and day 57 (boost vaccination). Descriptive participant demographics for the cohort are shown in **Table 1**.

**Table 1:** Demographics of participants receiving the experimental malaria vaccine regimen.

	Participants who received ChAd63 MVA ME-TRAP per-protocol (*n* = 336)
**Sex**	
Male	166 (49.4%)
Female	170 (50.6%)
**Age**	
Average (months)	10 (range: 5.0-17.3)
5-8 months	107 (31.8%)
9-12 months	120 (35.7%)
13-17 months	109 (32.4%)

Malaria infection was assessed by smear microscopy at the time of vaccination. 26.8% [90/336] of participants had detectable parasitaemia at either vaccination timepoint. The causative species identified in all infections was *P. falciparum* and geometric mean parasite density was 1823 [95% CI: 1303-2550] trophozoites/µl. Frequency of patent parasitaemia did not differ by gender, with 25.9% and 27.6% of males and females respectively having a positive microscopy result at either timepoint [*p* = 0.806]. The prevalence of parasitaemia increased with age. 15/107 [14%] of the 5-8 month age group had a positive microscopy result at either timepoint, compared to 36/120 [30%] and 39/109 [35.8%] of 9-12 month olds and 13-17 month olds respectively. The mean age of study participants was 9.9 [95% CI: 9.45-10.39] months and 11.4 [95% CI: 10.7-12.1] months in uninfected and infected infants respectively [*p* = 0.012].

### 3.2 Patent parasitaemia at vaccination is associated with reduced humoral but not cellular immunogenicity

**Figure 1:**
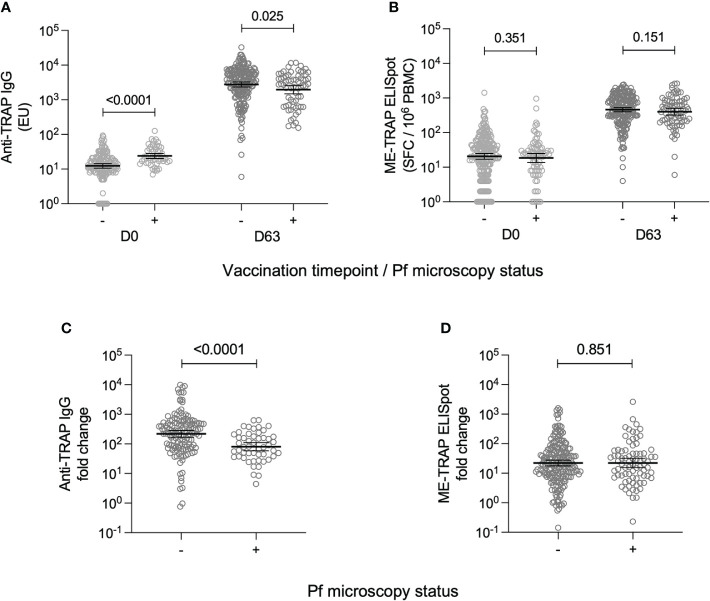
T cell and antibody responses to the vaccine antigen ME-TRAP, stratified by parasite smear microscopy status. **(A)** IgG titres against TRAP, measured at day 0 and day 63 (one week post-boost vaccination). Data are shown in standardised ELISA units. **(B)** *ex vivo* IFNγ ELISpot response to ME-TRAP, measured at day 0 and day 63. Data are shown in spot forming cells per 10^6^ peripheral blood mononeucleocytes. **(C)** Fold change in IgG titre against TRAP between day 0 and day 63. **(D)** Fold change in *ex vivo* IFNγ ELISpot response to ME-TRAP between day 0 and day 63. For all plots, - and + indicate individuals split by negative and positive *P. falciparum* smear microscopy results respectively. *p* values shown are Mann-Whitney tests. Lines and error bars denote geomeans and 95% confidence intervals.

Peak vaccine-specific immune responses at day 63 were compared between individuals who were parasite positive at either vaccination timepoint or negative throughout. At day 63, anti-TRAP IgG titres were significantly lower in participants who had a positive parasite blood smear [GMT: 1968; 95% CI: 1489-2600], compared to participants who were parasite-negative throughout [2775; 2347-3280] (**Figure 1A**). Conversely, anti-TRAP IgG titres were significantly higher at day 0 in the parasite positive group [24; 20.3-28.4] compared to the parasite negative group [12.4; 10.7-14.4]. This meant that day 0 to day 63 fold change was significantly reduced in participants with a positive blood smear result (**Figure 1C**).

It was assessed whether the timing or magnitude of parasite infection correlated with reduced humoral vaccine immunogenicity. There was no difference in anti-TRAP IgG titres at day 63 depending on whether the infection was at day 0, day 57 or both (**Supplementary Figure S1A**). There was also no significant association between the absolute parasite count in smear-positive volunteers and the anti-TRAP IgG titre at day 63 (**Supplementary Figure S1B**).

The effect of detectable parasitaemia at vaccination on cellular vaccine immunogenicity was also assessed. At day 0, there was no difference in *ex vivo* IFNγ ELISpot responses between parasite-positive [median: 24.0; 95% CI: 20.0-26.0] and parasite-negative volunteers [24.0; 20.0-32.0] (**Figure 1B**) At day 63, the median *ex vivo* IFNγ ELISpot response in parasite-positive volunteers was 457.0 [95% CI: 304.0 – 566.0] and in parasite negative volunteers was 525.0 [448.0-616.0], however this difference was not statistically significant (**Figure 1B**) As a result, there was also no significant difference in day 0 to day 63 fold change between parasite-positive and parasite-negative individuals (**Figure 1D**).

### 3.3 Anti-AMA-1 titre is associated with reduced cellular vaccine immunogenicity

Antibody titres to the blood-stage *P. falciparum* Apical Membrane Antigen 1 (AMA-1) were measured in sera at day 63 as a correlate of prior exposure to infection. Overall, 180/236 [76.3%] volunteers with an available sample had detectable levels of anti-AMA-1 IgG at day 63. The geometric mean titre was 33.6 [95% CI: 24.7-45.6] and this was not significantly different when stratified by sex or age group, however anti-AMA-1 IgG titres were significantly higher in the group with a positive smear microscopy result at vaccination [GMT: 209.0; 95% CI: 130.2-335.5], compared to the parasite-negative group [17.2; 12.4-24.0] (**Supplementary Figure S2**).

**Figure 2:**
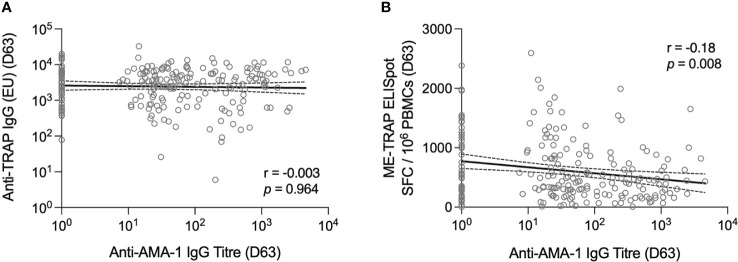
T cell and antibody responses to the vaccine antigen ME-TRAP, correlated with anti-AMA-1 IgG titres. **(A)** Anti-TRAP IgG titres measured at day 63 (one week post-boost vaccination) correlated with anti-AMA-1 IgG titres also measured at day 63. Data are shown in standardised ELISA units. **(B)** *ex vivo* IFNγ ELISpot response to ME-TRAP, measured at day 63 correlated with anti-AMA-1 IgG titres also measured at day 63. ELISpot data are shown in spot forming cells per 10^6^ peripheral blood mononeucleocytes and standardised ELISA units for anti-AMA-1 titres. Correlation was measured by Spearman r. Solid lines are simple linear regression with dotted lines showing 95% confidence intervals.

Anti-AMA-1 IgG titres were correlated against TRAP-specific IgG and T cell responses at day 63. No correlation was observed between anti-AMA-1 IgG and anti-TRAP IgG titres however, there was a weak negative association between the *ex vivo* IFNγ ELISpot response and anti-AMA-1 titre (**Figure 2**).

### 3.4 Vaccine immunogenicity is not associated with nutritional status

Weight-for-length Z scores (WLZ) were calculated at the screening visit using the standardised WHO international reference population (18). The median WLZ was -1.10 [range: 1.56 – -3.72]. 61/336 [18.2%] participants met the WHO criteria for moderate or severe malnourishment (WLZ < -2.0) and 0/336 participants were classified as overweight or obese (WLZ > 2.0). WLZ were not significantly different by gender, however they did vary by age. WLZ were significantly lower in the 9-12 month old group [median: -1.27; 95% CI: -1.13 – -1.61] compared to both the 5-8 month old group [median: -0.78; 95% CI: -0.53 – -1.08] and the 13-17 month old group [median: -1.10; 95% CI: -0.68 – -1.26] (**Supplementary Figure S3**).

**Figure 3:**
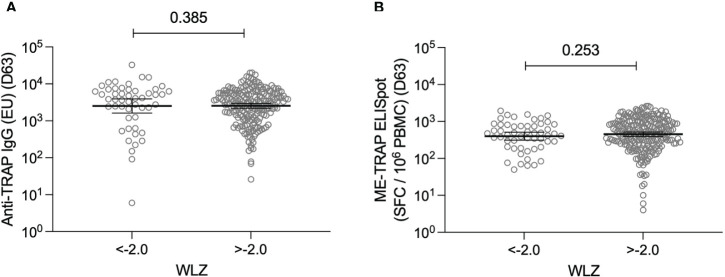
T cell and antibody responses to the vaccine antigen ME-TRAP, stratified by nutritional status. **(A)** Anti-TRAP IgG titres measured at day 63 (one week post-boost vaccination) stratified by weight-for-length Z scores <-2.0 and >-2.0. Data are shown in standardised ELISA units. **(B)** *ex vivo* IFNγ ELISpot response to ME-TRAP, measured at day 63 stratified by weight-for-length Z scores <-2.0 and >-2.0. Data are shown in spot forming cells per 10^6^ peripheral blood mononeucleocytes. WLZ <-2.0 suggests moderate or severe malnutrition. *p* values shown are Mann-Whitney tests. Lines and error bars denote geomeans and 95% confidence intervals.

WLZ were used to investigate a relationship between nutritional status and vaccine immunogenicity. Immune responses at day 63 were compared between participants who were classified as moderately or severely malnourished and those with a normal WLZ. No significant differences were observed for either antigen-specific IgG or T cell responses (**Figure 3**). Similarly, no significant relationships were observed when correlation analysis was performed instead (**Supplementary Figure S4**).

### 3.5 Vaccine efficacy against uncomplicated malaria is improved in uninfected individuals

**Figure 4:**
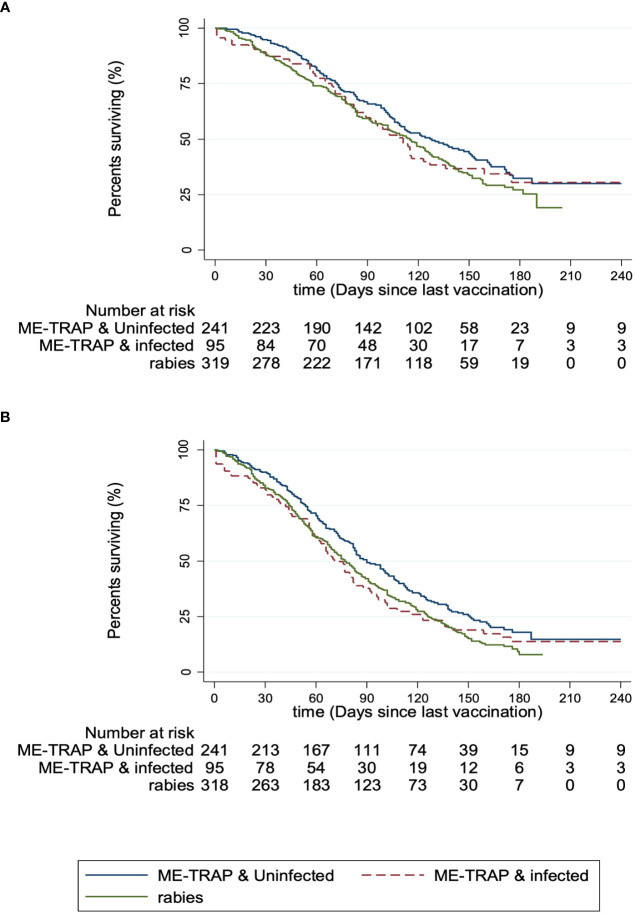
Protective efficacy against clinical malaria, stratified by parasite smear microscopy status. **(A)** Kaplan-Meier survival analysis for patients meeting the primary case definition for malaria (defined as fever (axillary temperature ≥37.5°C) with *P. falciparum* count > 5,000 trophozoites/µl of blood). **(B)** Kaplan-Meier survival analysis for patients meeting the secondary case definition for malaria (defined as fever (axillary temperature ≥37.5°C) with *P. falciparum* count > 0 trophozoites/µl of blood). Participants receiving the experimental vaccine regimen were stratified by *P. falciparum* smear microscopy status (ME-TRAP & uninfected/ME-TRAP & infected) and compared to participants receiving the rabies control vaccine regimen.

Finally, vaccine efficacy was calculated separately for volunteers with a parasite-negative or parasite-positive blood smear compared to the rabies vaccine control group. The incidence of clinical malaria meeting the primary case definition was 7.9 [95% CI: 6.8-9.2] and 9.9 [7.9-12.4] per thousand person-years in uninfected individuals and infected individuals respectively, compared to 10.0 [8.9-11.4] in the rabies vaccine control group. The Cox hazard ratio (HR) for vaccine efficacy in uninfected individuals compared to the rabies control group was 19.8% [95% CI: 0-35.7; *p* = 0.50] and in infected individuals was 3.3% [-12.5-16.8; *p* = 0.69] in adjusted analyses (**Figure 4A**). Using the secondary case definition, vaccine efficacy was 22.9% [95% CI: 6.5-36.4; *p* = 0.14] and 1.8% [-12.0-13.9; *p* = 0.58] in adjusted analyses for uninfected and infected individuals respectively (**Figure 4B**). Overall, this shows that although statistically significant efficacy was not demonstrated in either group compared to the rabies control group, the efficacy was increased by 83.3% and 92.1% using primary and secondary cases definitions respectively in uninfected compared with infected individuals.

## 4 Discussion

This study aimed to identify factors which may be associated with reduced immunogenicity of the candidate malaria vaccine ChAd63 MVA ME-TRAP in 5-17 months old infants and children in Burkina Faso. We previously published results showing the vaccine did not elicit significant efficacy against clinical malaria in this cohort despite promising results in adults (9). Because cellular immunogenicity was lower than that observed in adults and because malaria transmission was higher here than at other trial sites, it was hypothesised that malaria-induced immunosuppression may underlie some of the reduced effectiveness observed in this trial. Similarly, malnutrition may also contribute to dampened immune responses to vaccination. A role for both malaria infection and malnutrition in impaired immunity is widely accepted (14, 19), however few studies have directly assessed their impact on response to vaccination.

We assessed exposure to malaria by two methods. Firstly, blood smears were taken at vaccination timepoints to assess any association with acute concurrent malaria infection at the point of vaccination. IgG titres against the blood-stage *P. falciparum* antigen, AMA-1, were also measured. Whilst anti-AMA-1 IgG titres were also elevated in smear-positive individuals, it is known that antibodies against blood-stage antigens are cumulatively acquired over repeated infections and it has previously been shown that they broadly correlate with intensity of prior exposure (20, 21). Using anti-AMA-1 titres as a surrogate marker of prior exposure intensity may identify associations with chronic, repeated malaria infections.

In the data presented here, we show that the antibody response one week after the boost is reduced in individuals who had patent parasitaemia at vaccination, whilst the T cell response measured by *ex vivo* IFNγ ELISpot was unaffected. We also observed that the titre of anti-AMA-1 IgG is negatively correlated with peak T cell response to vaccination, but not the antibody response. This might suggest that acute malaria infection could reduce humoral immunity to vaccination with ChAd63 MVA ME-TRAP, whilst chronic parasite exposure may impact cell mediated immunity.

The vaccine tested in this study was previously tested in Phase IIb studies in adults in Kenya (10) and Senegal (22). Interestingly, vaccine immunogenicity and efficacy were significantly higher in the Kenyan study than in Senegal, however, the PCR prevalence of malaria infection was higher in Kenyan participants at the time of vaccination. It is currently unknown whether PCR positivity at the time of vaccination was directly associated with subsequent vaccine responses to viral vectored ME-TRAP and if so, why these results might differ to our findings presented here. One potential major difference to explore is how infant responses to vaccination compare to adults whose immune responses have been modulated by years of repeated exposures to malaria infection. Future work should focus on expanding our analysis to other trials and investigating differences identified between different population groups.

The effect of malaria infection on the suppression of responses to other vaccines has previously been reviewed (12), however there are few other studies with comparable data with which to contextualise these results. A previous iteration of the ME-TRAP vaccine using the attenuated fowlpox strain, FP9 in a prime-boost regimen with MVA, has also been assessed for the impact of malaria infection on its immunogenicity (13). This study was conducted in 1-6 year olds in Kenya. Interestingly, it showed reduced *ex vivo* IFNγ ELISpot responses in vaccinees who were positive by smear microscopy at the time of vaccination. This outcome may reflect a difference in how FP9 induces immune responses compared to ChAd63, a difference due to the different age ranges between studies or a difference due to either environmental or genetic factors between the study populations. Antibody titres were not assessed in this study. Few other studies have similarly measured the effect of chronic malaria exposure on vaccine responses, making it difficult to compare this data to the literature.

A limitation of using smear microscopy to diagnose malaria infection is its poorer sensitivity compared to molecular methods such as qPCR (23, 24). Some submicroscopic infections may have been misclassified as parasite-negative in this analysis. These may have been relevant considering the complex immune mechanisms involved in maintaining low-density asymptomatic parasitaemia (25). A disadvantage of using anti-AMA-1 titres as a surrogate of intensity of exposure is that this may be confounded by an independent immunological factor which also affects vaccine immunogenicity. Titres may also be skewed by current infection. Ideally, documented episodes of malaria infection would have been used to estimate prior exposure intensity, however this was not possible in this retrospective analysis and would be logistically complex.

Efficacy was recalculated by stratifying volunteers in the experimental vaccine arm by parasite smear microscopy status. Although no significant efficacy compared to the control rabies vaccine arm was observed, the efficacy appeared comparably higher for uninfected vaccinees. Recently, it was shown that a significant increase in efficacy occurred when parasitaemia was cleared before *P. falciparum* sporozoite (PfSPZ) vaccination in a controlled human malaria infection model (26). This suggests blood-stage parasitaemia may limit the response to some pre-erythrocytic malaria vaccines. The data presented in our analysis should be interpreted cautiously since the trial was not initially powered to detect differences between sub-groups and because efficacy was low overall.

Overall, these data suggest a potential association between malaria infection and sub-optimal response to vaccination with ChAd63 MVA ME-TRAP. This warrants further investigation, for example, through testing the effect of malaria chemoprophylaxis before vaccination on subsequent vaccine responses. It also highlights the need to continue investigating the complex mechanisms involved in malaria-induced immunosuppression. Various mechanisms have been proposed which might explain how malaria infection impacts responses to heterologous antigens. These include inflation of the CD4+ regulatory T cell compartment (27, 28), phenotypic exhaustion of B and T cells (29–31) and induction of phenotypically sub-optimal T follicular helper cells (32) and B cells (33). In the future it would be relevant to investigate the function of these cell subsets in the context of responses to malaria vaccination. Understanding the mechanism of malaria-induced immunosuppression might help to inform the design of vaccines which can bypass these effects. Similarly, it will be important to understand the impact of other chronic immunomodulatory infections common in this region such as helminth infection (34) and viral infections including HIV, cytomegalovirus and Epstein Barr Virus.

Nutritional status, another factor with a potential role in immunosuppression, was assessed using weight-for-length Z-scores, as recommended by the WHO for assessing acute malnutrition in infants aged <60 months. No associations were seen between WLZ and T cell or antibody titres against ME-TRAP, suggesting nutritional status may not affect immunogenicity in this specific context. However, it should be noted that the dataset was skewed to below the international average WLZ indicating that overall, the study population was underweight. Despite this, there were relatively few children with a WLZ < -3, meeting the definition for severe acute malnourishment. This study may have been underpowered to detect differences across a representative range of WLZ and nutritional statuses. All participants who met the eligibility criteria for inclusion also presented without clinical signs of marasmus or kwashiorkor. Therefore, an effect in clinically unwell, severely malnourished children cannot be ruled out. The effect of malnutrition in infancy on vaccine responses has been previously reviewed (15). Data in this field remains insufficient, however whilst most studies show malnourished infants do seroconvert to all routinely administered vaccines, there is some evidence of reduced antibody titres against diphtheria, tetanus and pertussis (DTP) vaccine (35). More evidence is required on the effect of malnutrition on T cell responses to vaccination as well as for vaccination using viral vectored platforms, particularly given the widespread use of these viral vectors for COVID-19 vaccines.

In summary, we have shown that *P. falciparum* infection, but not nutritional status, is negatively associated with the immunogenicity of an experimental malaria vaccine ChAd63 MVA ME-TRAP, and that this may result in reduced vaccine efficacy. Separate mechanisms in acute and chronic infection may exist impacting humoral and T cell immunity, respectively. Further work to explore these mechanisms is warranted to develop improved strategies for vaccination against malaria. Because the mechanisms by which malaria suppresses immune responses are likely broad and non-specific, these findings may have relevance for many other vaccines being tested and rolled-out in regions with a high burden of infectious diseases including malaria, as well as malnutrition.

## Data availability statement

The raw data supporting the conclusions of this article will be made available by the authors, without undue reservation.

## Ethics statement

The studies involving human participants were reviewed and approved by Centre National de Recherche et de Formation sur le Paludisme (CNRFP) institutional bioethics committee. Ministry of Health Ethical Committee for Biomedical Research (Burkina Faso). Oxford Tropical Research Ethics Committee (OXTREC) (University of Oxford). Written informed consent to participate in this study was provided by the participants’ legal guardian/next of kin.

## Author contributions

SS, AT, AH, KE, AO, NV, and IN developed the clinical trial protocol. SS, AT, AH, KE, AO, AD, NV, and IN were involved with conducting the clinical trial. AT, RM, and OH performed the laboratory procedures and data analysis presented here in collaboration with SS and KE. AT and RM drafted the original manuscript which was edited by SS, AH, KE, NV, AO, AD, OH, and IN. All authors read and approved the final manuscript.

## Funding

This work was supported by an award from the European and Developing Countries Clinical Trials Partnership (EDCTP) and was performed by the Malaria Vectored Vaccines Consortium (MVVC), (Grant number IP.2008.31100.001). The European Vaccine Initiative (EVI) was the coordinator of the EDCTP-funded MVVC project. Co-funding was provided by the Swedish International Development Cooperation Agency (SIDA), the UK Medical Research Council (MRC), the Austrian Federal Ministry of Science and Research, and Irish Aid, Department of Foreign Affairs and Trade, Ireland. RM was supported by a PhD studentship from the Wellcome Trust (Grant number 109026/Z/15/Z). The funders had no role in study design, data collection and analysis, decision to publish, or preparation of the manuscript.

## Acknowledgments

We thank the CNRFP staff at the Banfora research unit for their collaboration and Ceri McKenna for study monitoring. We are thankful to the Data Safety Monitoring Board and all the study volunteers. We particularly note the contribution of the late Dr Egeruan Babatunde Imoukhuede to this work.

## Conflict of interest

AH is a named inventor on patent applications and issued patents relating to malaria vectored vaccines and immunization regimes.

The remaining authors declare that the research was conducted in the absence of any commercial or financial relationships that could be construed as a potential conflict of interest.

## Publisher’s note

All claims expressed in this article are solely those of the authors and do not necessarily represent those of their affiliated organizations, or those of the publisher, the editors and the reviewers. Any product that may be evaluated in this article, or claim that may be made by its manufacturer, is not guaranteed or endorsed by the publisher.
