# Supplementary material for: Impact of exposure to malaria and nutritional status on responses to the experimental malaria vaccine ChAd63 MVA ME-TRAP in 5-17 month-old children in Burkina Faso
Source: Front Immunol. 2022 Dec 2;13:1058227. doi: 10.3389/fimmu.2022.1058227 (PMC9755991; doi:10.3389/fimmu.2022.1058227)
Supplement: Supplementary file 1 [file DataSheet_1.pdf]

**Supplementary figure S1**

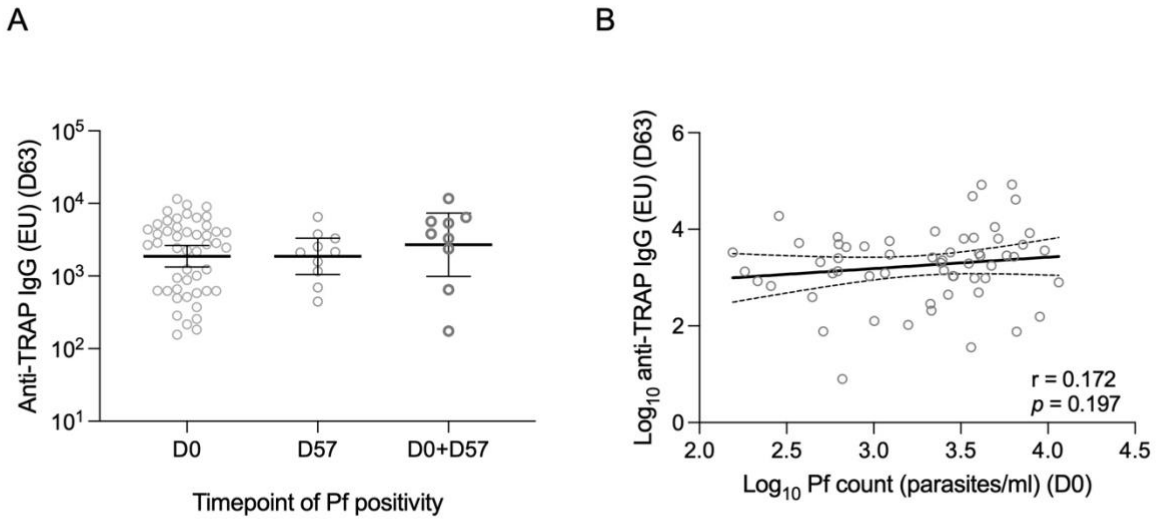


**(A)** Anti=TRAP IgG titre measured at day 63 in participants with a positive *P. falciparum* blood smear, split by the timepoint at which the test was positive. Lines and error bars indicate geomeans and 95% confidence intervals. **(B)** Correlation between anti-TRAP IgG titre measured at day 63 in participants with a positive *P. falciparum* blood smear at day 0, correlated with the parasite count. Correlation measured by Spearman r. Solid lines are simple linear regression with dotted lines showing 95% confidence intervals.

**Supplementary figure S2**

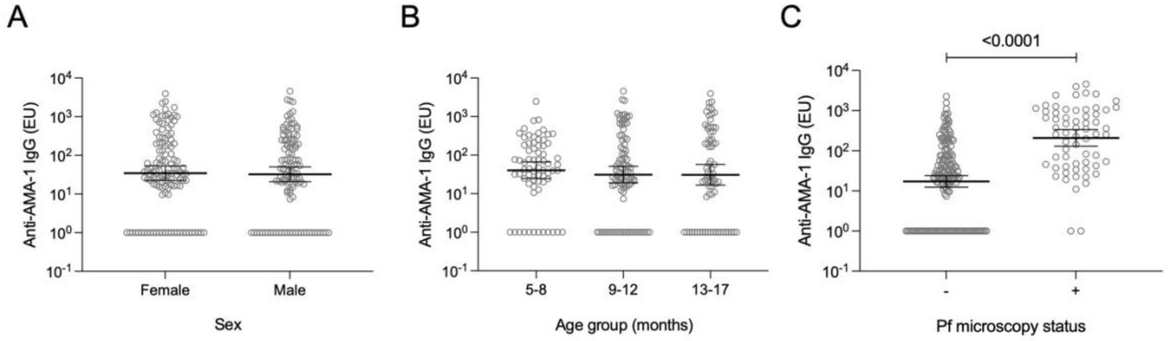


Anti-AMA-1 IgG titres measured at day 63, stratified by **(A)** sex, **(B)** age, and **(C)** *P. falciparum* blood smear status. *p* values are from Mann-Whitney tests. Lines and error bars indicate geomeans and 95% confidence intervals.

**Supplementary figure S3**

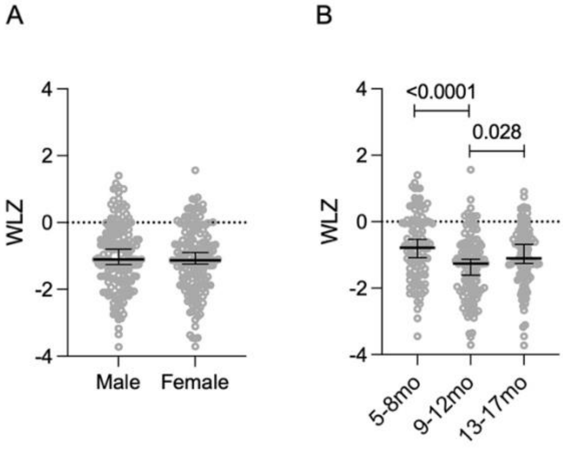


Weight-for-length Z scores stratified by **(A)** sex, and **(B)** age. *p* values are from Kruskal-Wallis testing. Lines and error bars indicate geomeans and 95% confidence intervals.

**Supplementary figure S4**

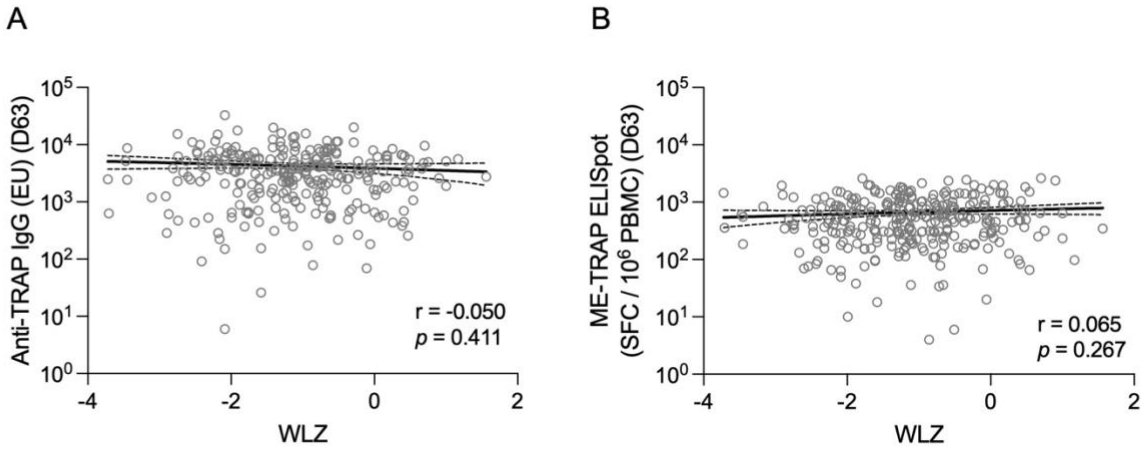


Weight-for-length Z scores correlated with **(A)** anti-TRAP IgG titres measured at day 63 in standardised ELISA units, and **(B)** *ex vivo* IFNγ ELISpot response to ME-TRAP, measured day 63 in spot forming cells per $10^6$ peripheral blood mononucleocytes. Correlation measured by Spearman r. Solid lines are simple linear regression with dotted lines showing 95% confidence intervals.

**A Phase 1/2b double blind randomised controlled trial of the efficacy, safety and immunogenicity of heterologous prime-boost immunisation with the candidate malaria vaccines ChAd63 ME-TRAP and MVA ME-TRAP in 5-17 month old Burkinabe infants and children**

| | |
|---|---|
| Study reference: | VAC050 |
| Protocol Version: | 2.3 |
| Date: | 18th July 2014 |
| OXTREC Number: | 41-12 |
| Sponsor: | University of Oxford |
| Funding body: | EDCTP |
| Author(s): | **CNRFP:** Sodiomon B. Sirima, Alfred B. Tiono, Issa N. Ouédraogo<br>**UOXF:** Nick Anagnostou, Susanne Sheehy, Alison Lawrie, Rachel Roberts, Adrian VS Hill |

 © University of Oxford, 2013

**Statement of Compliance**

The trial will be conducted in compliance with the protocol, International Conference on Harmonisation Good Clinical Practice Guideline E6 (R1) (ICH-GCP) and the applicable regulatory requirements.

**Signatures**

"I have read this protocol and agree to abide by all provisions set forth therein. I agree to comply with the principles of the Declaration of Helsinki and the International Conference on Harmonisation Tripartite Guideline on Good Clinical Practice."

PROTOCOL SIGNATURE SHEET

Principal Investigator:
________________________________ Date:

**Confidentiality Statement**
This document contains confidential information that must not be disclosed to anyone other than the Sponsor, the Investigator Team, members of the Independent Ethics Committee and the Burkina regulatory authority. This information cannot be used for any purpose other than the evaluation or conduct of the clinical investigation without the prior written consent of Professor Adrian Hill.

 © University of Oxford, 2013

## CONTENTS

Statement of Compliance 2
Signature Page 2
Confidentiality statement 2
Contents 3
Key Roles and General Information 4
List of Abbreviations 7
1. Study synopsis 9
2. Background Information 15
3. Rationale 24
4. Objectives 26
5. Description and Justification of Study Design 28
6. Inclusion and Exclusion Criteria 36
7. Investigational Medicinal Products 38
8. Study Schedule and Procedures 40
9. Assessment of Scientific Objectives 52
10. Safety reporting 55
11. Data Handling and Record Keeping 60
12. Data access and quality assurance 62
13. Ethical considerations 63
14. Indemnity / Compensation / Insurance 64
15. References 65

 © University of Oxford, 2013

## KEY ROLES AND GENERAL INFORMATION

| | |
|---|---|
| **Trial Centre:** | Centre National de Recherche et de Formation sur le Paludisme (CNRFP)/ Unité de Recherche Clinique de Banfora (URC-B)<br>01 BP 2208 Ouagadougou 01<br>Burkina Faso |
| **Principal Investigator:** | Dr Sodiomon B Sirima<br>Centre National de Recherche et de Formation sur le Paludisme (CNRFP)/ Unité de Recherche Clinique de Banfora (URC-B)<br>01 BP 2208 Ouagadougou 01<br>Burkina Faso |
| **Study Physician** | Dr Jean Baptist B. Yaro |
| **Co-Investigator(s):** | ***Centre National de Recherche et de Formation sur le Paludisme (CNRFP)/ Unité de Recherche Clinique de Banfora (URC-B)***<br>— Dr Alfred B. Tiono<br>— Dr Issa Nébié Ouédraogo<br>— Dr David T. Kangoué<br>— Dr Edith C. Bougouma<br>— Dr André Lin Ouédraogo<br>— Dr Issiaka Soulama<br><br>01 BP 2208 Ouagadougou 01<br>Burkina Faso<br>***United Kingdom (Jenner Institute, Nuffield Dept. of Clinical Medicine, University of Oxford)***<br>— Prof Adrian Hill<br>— Dr Susanne Sheehy<br>— Dr Nicholas Anagnostou<br>— Dr Alison Lawrie<br>— Dr Rachel Roberts<br>— Dr Philip Bejon (statistician)<br><br>Centre for Clinical Vaccinology and Tropical Medicine<br>University of Oxford Churchill Hospital, Oxford OX3 7LE |

 © University of Oxford, 2013

**Local Safety Monitor:**
Dr DAO Fousséni
Paediatrician National Teaching Hospital Yalgado Ouédraogo
03 BP 7022 Ouagadougou 03
Burkina Faso

**Data Safety and Monitoring Board:**
Chair: Professor Geoffrey Targett
Emeritus Professor of Immunology of Parasitic Diseases
London School of Hygiene & Tropical Medicine
Room 410, Keppel Street, London WC1E 7HT, UK

DSMB members:
Dr. Paul Milligan
London School of Hygiene & Tropical Medicine
Reader in Epidemiology and Medical Statistics
Room 411a, Keppel Street, London WC1E 7HT, UK

Professor Mahamadou Thera
MRTC, Bamako, University of Mali
Colline de Badalabougou
BP E2528, Mali

Dr. Bernhards Ogutu
KEMRI
P.O. Box 2254 - 00202
Nairobi
Kenya

**Project Managers (Oxford):**
Dr Alison Lawrie,
Ms Rachel Roberts

Centre for Clinical Vaccinology and Tropical Medicine
University of Oxford Churchill Hospital, Oxford OX3 7LJ

**Sponsor:**
University of Oxford
Named contact: Heather House
Clinical Trials and Research Governance
Manor House
John Radcliffe Hospital
Oxford, OX3 9DZ
Tel: 01865 743004
Fax: 01865 743002
Email: *heather.house@admin.ox.ac.uk*

**External Monitor:**
Ceri McKenna

 © University of Oxford, 2013

Appledown Clinical Research Limited
Orchard's End,
Greenland's Lane,
Prestwood,
Gt. Missenden,
Bucks. HP16 9QX

 © University of Oxford, 2013

## List of abbreviations

| | |
|---|---|
| **ChAd63** | Chimpanzee Adenovirus 63 |
| **AE** | Adverse event |
| **ALT** | Alanine aminotransferase |
| **BCG** | Bacille Calmette-Guerin |
| **CCVTM** | Clinical Centre for Vaccinology and Tropical Medicine |
| **CBF** | Clinical Biomanufacturing Facility |
| **ChAd** | Chimpanzee-derived adenovirus (also abbreviated AdCh) |
| **CMI** | Cell-mediated immunity |
| **CRF** | Case report form |
| **CTL** | Cytotoxic T lymphocytes |
| **DSMB** | Data Safety and Monitoring Board |
| **DTP** | Diphtheria-Tetanus-Pertussis Vaccine |
| **EDCTP** | European and Developing Countries Clinical Trials Partnership |
| **ELISA** | Enzyme-linked immunosorbant assay |
| **ELISPOT** | Enzyme-linked immunospot |
| **EPI** | Expanded Programme on Immunization |
| **FBC** | Full blood count |
| **FP9** | Fowlpox 9 |
| **GCP** | Good Clinical Practice |
| **GMP** | Good Manufacturing Practice |
| **HepB** | Hepatitis B (vaccine) |
| **Hib** | Haemophilus Influenzae type b |
| **IB** | Investigator’s Brochure |
| **ICS** | Intracellular Cytokine Staining |
| **IDT** | Impfstoffwerk Dessau-Tornau GmbH |
| **IEC** | Independent Ethics Committee |
| **IFN-γ** | Gamma interferon |
| **IMP** | Investigational Medicinal Product |
| **IRB** | Independent Review Board |
| **IPT** | Intermittent preventive antimalarial therapy |
| **HBV** | Hepatitis B virus |
| **HIV** | Human immunodeficiency virus |
| **HLA** | Human leukocyte antigen |
| **LFT** | Liver function test |
| **LSM** | Local safety monitor |
| **ME-TRAP** | Multiple epitope string with thrombospondin-related adhesion protein |
| **MHRA** | Medicines and Healthcare products Regulatory Agency |
| **MVA** | Modified vaccinia Virus Ankara |
| **MVVC** | Malaria vectored vaccine consortium: an EDCTP funded research collaboration |

 © University of Oxford, 2013

| | |
|---|---|
| **NHS** | National Health Service |
| **OXTREC** | Oxford Tropical Research Ethics Committee |
| **PBMC** | Peripheral blood mononuclear cells |
| **PCR** | Polymerase chain reaction |
| **pfu** | Plaque forming units |
| **SAE** | Serious adverse event |
| **SC** | Subcutaneous |
| **SOP** | Standard Operating Procedures |
| **SUSAR** | Suspected unexpected serious adverse reaction |
| **vp** | Virus particles |


© University of Oxford, 2013

## 1. Study synopsis

| | |
|---|---|
| **Trial Title** | **A Phase 1/2b double blind randomised controlled trial of the efficacy, safety and immunogenicity of heterologous prime-boost immunisation with the candidate malaria vaccines ChAd63 ME-TRAP and MVA ME-TRAP in 5-17 month old Burkinabe infants and children** |
| **Trial Identifier** | VAC 050 |
| **Clinical phase** | I/IIb |
| **Investigational medicinal products** | |
| *Active ingredients* | Chimpanzee Adenovirus 63 expressing multiple epitopes with thrombospondin-related adhesion protein (ChAd63 ME-TRAP)<br><br>Modified vaccinia Virus Ankara expressing multiple epitopes with thrombospondin-related adhesion protein (MVA ME-TRAP) |
| *Finished products and doses* | ChAd63 ME-TRAP: 5 x $10^{10}$vp<br>MVA ME-TRAP: 1 x $10^{8}$ pfu |
| *Route of administration* | Intramuscular needle injection into the anterolateral thigh |
| **Principal Investigator** | Dr Sodiomon B. Sirima |
| **Trial Centre** | Centre National de Recherche et de Formation sur le Paludisme (CNRFP)/<br>Unité de Recherche Clinique de Banfora (URC-B)<br>01 BP 2208 Ouagadougou 01<br>Burkina Faso |
| **Planned Trial Period** | November 2012 until May 2015 |
| **Study Duration** | 30 months |
| **Subject Duration** | 26 months from Day 0 |
| **Objectives: Lead-in Safety Evaluation** | To assess the safety of ChAd63 ME-TRAP / MVA ME-TRAP prime-boost immunisation in a cohort of 5-17 month old Burkinabe infants and children |
| **Objectives: Phase 2b Trial** | |
| *Primary Objective* | To assess the protective efficacy against clinical malaria of ChAd63 ME-TRAP / MVA ME-TRAP prime-boost immunisation, in 5-17 month old infants and children living in a malaria-endemic area, for 6 months after the last vaccination |

 © University of Oxford, 2013

| | |
|---|---|
| *Secondary Objectives* | *<u>Duration of Protective efficacy against clinical malaria</u>*<br>To assess the protective efficacy against clinical malaria of ChAd63 ME-TRAP / MVA ME-TRAP prime-boost immunisation, in 5-17 month old infants and children living in a malaria-endemic area, for 12 and 24* months after the last vaccination.<br><br>*<u>Efficacy against asymptomatic P. falciparum infection</u>*<br>To assess the protective efficacy against asymptomatic *P. falciparum* infection of ChAd63 ME-TRAP / MVA ME-TRAP prime-boost immunisation, in 5-17 month old infants and children living in a malaria-endemic area, 6, 12 and 24* months after the last vaccination<br><br>*<u>Efficacy against secondary case definitions of clinical malaria</u>*[+]<br>To assess the protective efficacy against secondary case definitions of clinical malaria of ChAd63 ME-TRAP / MVA ME-TRAP prime-boost immunisation, in 5-17 month old infants and children living in a malaria-endemic area, for 6, 12 and 24* months after the last vaccination<br><br>*<u>Safety Objective</u>*<br>To assess the safety and reactogenicity of ChAd63 ME-TRAP / MVA ME-TRAP heterologous prime-boost immunisation, in 5-17 month old infants and children living in a malaria-endemic area, for 6, 12 and 24 months after the last vaccination.<br><br>*<u>Immunogenicity Objectives</u>*<br>To assess the immunogenicity of ChAd63 ME-TRAP / MVA ME- TRAP heterologous prime-boost immunisation, in 5-17 month old infants and children living in a malaria-endemic area.<br><br>To explore the immunologic correlates of protective efficacy of ChAd63 ME-TRAP / MVA ME-TRAP prime-boost immunisation, in 5-17 month old infants and children living in a malaria-endemic area. |
| *Exploratory Objective* | *<u>Efficacy against incident cases of severe malaria</u>*<br>To assess the protective efficacy against severe malaria of ChAd63 ME-TRAP / MVA ME-TRAP prime-boost immunisation, in 5-17 month old infants and children living in a malaria-endemic area, for 6, 12 and 24* months after the last vaccination. |
| **Population** | Healthy Burkinabe infants and children aged 5 to 17 months at enrolment |
| **Planned Sample** | Phase 1 Lead-in Safety Evaluation: 30 participants<br>Phase 2b trial: 700 participants |
| **Vaccination Schedule** | |
| *Phase 1 Lead-in Safety Evaluation* | ChAd63 ME-TRAP on Day 0, and MVA ME-TRAP on Day 56 |

 © University of Oxford, 2013

| | |
|---|---|
| *Phase 2b Trial* | ChAd63 ME-TRAP on Day 0, and MVA ME-TRAP on Day 56; or Rabies vaccine on Day 0 and Day 56. |
| **Follow-up duration** | 26 months from Day 0 |
| **Blood Sampling Schedule** | Phase I Lead-in Safety Evaluation: Screening, Days 0, Day 21, Day 56, Day 63, 243, 423, 783<br><br>Phase 2b trial: Screening, Day 0$^+$ , Day 21, Day 63, Day 243, Day 423, Day 783<br><br>$^+$: nested cohort of participants only |
| **Primary Evaluation Endpoint: Lead-in Safety Evaluation**<br><br>**Primary Evaluation Endpoint: Phase 2b Trial** | Description of local and systemic solicited and unsolicited adverse events considered possibly, probably or definitely related to vaccination with ChAd63 ME-TRAP and MVA ME-TRAP<br><br>Time to first episode of malaria meeting the primary case definition of clinical malaria episode$^{\pm}$ over a period of 6 months of follow-up after the last vaccination (i.e., from study day 63 to study day 243). |
| **Endpoints: Phase 2b Trial** | ***Efficacy endpoints***<br><br><u>The following endpoints, as defined in Section 5 (Description and Justification of Study Design), subsection, Endpoints for the Phase 2b trial.</u><br>- *Primary case definition of clinical malaria episode*<br>- *Secondary case definitions of clinical malaria episode*<br>- *Primary case definition of asymptomatic P. falciparum infection*<br>- *Primary case definition of severe malaria*<br>- *Secondary case definitions of severe malaria*<br><br>***Safety endpoints***<br><br>SAEs occurring from first vaccination until the end of the study<br><br>Local and systemic solicited and unsolicited adverse events, considered possibly, probably, or definitely related to vaccination, occurring from first vaccination until 1 month post second vaccination (study day 93).<br><br>***Immunogenicity endpoints***<br><br>Immune responses and their determinants. This may include:<br>▪ T cell enumeration and characterisation, using ELISPOT, and flow cytometry with intracellular cytokine staining<br>▪ Measurement of antibodies to TRAP and other malaria antigens, using ELISA<br>▪ Measurement of antivector immune responses<br>▪ Enumeration of antibody-secreting cells, using ELISPOT and flow |

 © University of Oxford, 2013

cytometry
- Measurement of antibody response to other EPI vaccines administered to the participants
- Cytokine quantification in serum, using ELISA
- Evaluation of genetic determinants of clinical outcome and immune responses to TRAP, other malaria antigens and EPI vaccines using HLA typing, DNA and RNA analysis of polymorphisms and transcript levels, detection of haemoglobin gene variants, and other suitable methods
- Transcriptional profiling

| | |
|---|---|
| **Study Design** | Double-blinded, randomized controlled study, with an open-label lead-in safety evaluation |

* Depending on the outcome of the efficacy analysis of the 12 month data, this may be extended to 24 months following completion of vaccination

**Schematic of Study Design**

*Stage 1*

| Group Code | Age of subjects at first vaccination | Number of subjects | First vaccination on Day 0 | Second vaccination on Day 56 |
|---|---|---|---|---|
| Group A | 5 to 17 months | 30 | ChAd63 ME-TRAP $5 \times 10^{10}$vp | MVA ME-TRAP $1 \times 10^{8}$ pfu |

*Stage 2*

| Group | Age of subjects at first vaccination | Number of subjects | First vaccination on Day 0 | Second vaccination on Day 56 |
|---|---|---|---|---|
| Group B | 5 to 17 months | 350 | AdCh63 ME-TRAP $5 \times 10^{10}$vp | MVA ME-TRAP $1 \times 10^{8}$ pfu |
| Group C | 5 to 17 months | 350 | Rabies vaccine | Rabies vaccine |



 © University of Oxford, 2013

**Table 1: Timeline of study visits and procedures for participants in Groups A, B and C**

| Study Visit number | S | 1 | 2-4 | 5 | 6 | 7 | 8-10 | 11 | 12 | 13-16 | 17 | 18-22 | 23 | 24-34 | 35 |
|---|---|---|---|---|---|---|---|---|---|---|---|---|---|---|---|
| Clinic Visit | X | X | | X | X | X | | X | X | | X | | X | | X |
| Home visit | | | X | | | | X | | | X | | X | | X | |
| Day of Visit ** | D-30 to D-1 | D0 | D1, D2, D3 | D7 | D21 | D56 | D57, 58, 59 | D63 | D93 | D123, 153, 183, 213 | D243 | D273, 303, 333, 363, 393 | D423 | Days 453,483, 513,543, 573,603, 633,663, 693,723, 753 | D783 |
| Window Period | | | | -3/+7 | -6/+14 | ±14 | | -1/+7 | ±2 | ±14 | ±14 | ±14 | ±14 | ±14 | ±14 |
| Vaccination (Group A) | | ChAd63 ME-TRAP | | | | MVA ME-TRAP | | | | | | | | | |
| Vaccination (Group B, C) | | ChAd63 ME-TRAP or Rabies | | | | MVA ME-TRAP or Rabies | | | | | | | | | |
| Inclusion, Exclusion criteria | X | X | | | | X | | | | | | | | | |
| Informed consent | X | | | | | | | | | | | | | | |
| Medical history | X | (X) | | (X) | (X) | (X) | | (X) | (X) | (X) | (X) | (X) | (X) | (X) | (X) |
| Physical examination | X | (X) | | (X) | (X) | (X) | | (X) | (X) | (X) | (X) | (X) | (X) | (X) | (X) |
| Review contraindications to vaccination | | X | | | | X | | | | | | | | | |
| Recording of concomitant medication | X | X | X | X | X | X | X | X | X | X | X | X | X | X | X |
| Recording of solicited and unsolicited adverse events | | X | X | X | X | X | X | X | X | | | | | | |
| Recording of SAEs | | X | X | X | X | X | X | X | X | X | X | X | X | X | X |
| Blood Film for *P. falciparum* | X | | | X | | X | | X | | | X | | X | | X* |
| Blood Film for *P. falciparum* if axillary temp ≥37.5 and/or history of fever within last 24 hours | | X | X | X | X | X | X | | X | X | | X | | X | |
| Blood sampling | X | $X^{++}$, $X^{+}$ | | | X | $X^{++}$ | | X | | | X | | X | | X |



 © University of Oxford, 2013

** Each study visit will occur the indicated number of days from Day 0, within the window period for that visit

S: Screening Visit; X: procedure takes place, (X): procedure takes place as required at the discretion of the investigators; D : Day.

* if efficacy analysis is extended to include data to 24 months following completion of vaccinations

+: nested cohort of Group B and C volunteers only
++: Group A volunteers only

 © University of Oxford, 2013

# 2. Background Information

## 2.1 Introduction

### *Impact of malaria and the need for a vaccine*

Malaria is the preeminent tropical infectious disease globally, with a devastating effect on human health and society. There is estimated to have been approximately 250 million cases of malaria worldwide in 2008, mostly in Africa[1]. Approximately one million persons died, predominantly children under the age of five years[1]. Over 90% of cases were due to the malaria species, *Plasmodium falciparum*[1]. The enormous economic and social consequences of malaria have been well documented[2]. Malaria remains a potentially fatal hazard for travellers visiting malaria-endemic regions.

The development of a vaccine against malaria is a high priority and of great importance in the context of coordinated efforts to reduce the burden of malaria. Examples of the limitations of other measures aimed at reducing the burden of malaria include the development of resistance of Anopheles mosquitoes to certain insecticides; the development of resistance of malaria parasites to chemotherapeutic agents[4]; the absence of a gametocytocidal drug suitable for mass administration[5], and the risk of reimportation of malaria into geographic regions previously cleared of malaria using environmental elimination measures. The Roll Back Malaria (RBM) Partnership was launched in 1998 by the World Health Organization (WHO), the United Nations Children's Fund (UNICEF), the United Nations Development Programme (UNDP) and the World Bank. A major goal of the RBM Partnership is to support the development of a vaccine against malaria as a key future strategy for reducing mortality from malaria. The development of an effective vaccine is considered necessary for the global eradication of malaria[3].

### *Lifecycle of Plasmodium falciparum*

The lifecycle of *P. falciparum* is complex with stages in both human and mosquito hosts (Figure 1). The bite of infected female Anopheles mosquitoes transmits malaria sporozoites to the human host where they travel via the bloodstream to the liver and invade hepatocytes. Here, during the liver stage, they mature into merozoites for 6 to 7 days. Malaria parasites are not detectable in the blood stream during the liver stage. The hepatocytes then rupture, releasing a large number of merozoites into the bloodstream. Thus begins the blood stage. Merozoites invade erythrocytes where they multiply and after 2 days cause the erythrocyte to rupture, releasing progeny merozoites that in turn invade new erythrocytes. A small percentage of merozoites differentiate into gametocytes, which when ingested by a mosquito, unite with another gametocyte to create a zygote. The zygote matures and releases sporozoites which migrate to the mosquito's salivary glands and are

 © University of Oxford, 2013

injected into the human when the mosquito feeds. Patency refers to the ability to detect parasites on examination of the peripheral blood during the blood stage.

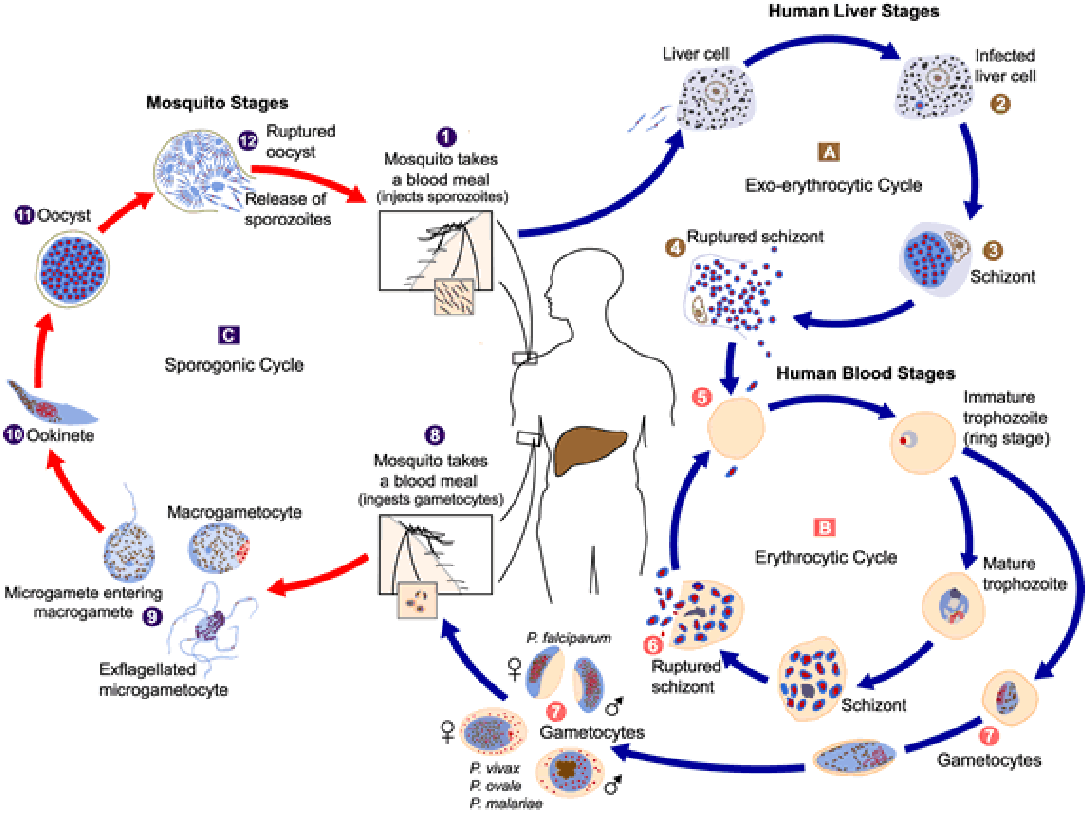


**Figure 1** *Lifecycle of Plasmodium falciparum*

***Progress towards a P. falciparum vaccine***

The candidate malaria vaccine, RTS,S, targets antibodies against the circumsporozoite protein (CS), which is expressed by the sporozoite at the preerythrocytic stage. Formulated with adjuvant, preliminary estimates of efficacy against infection after curative anti-malarial treatment were 34.0% (95%CI 8-53%) in adults[6] and 65.9% (95%CI 43-80) in infants[7]. Efficacy against the more clinically relevant endpoint of clinical malaria in 1-4 year old children was 29.9% (95%CI 11-45%) [8] . Efficacy against clinical malaria with the more immunogenic AS01E adjuvant was 52.9% (95%CI 28-69%) in children in Kenya and Tanzania over 8 months of follow-up and 39.2% over 12 months of follow-up[8,69]. A Phase III trial with RTS,S/AS01E is currently in progress. Preliminary findings[70] show efficacy against severe malaria of 34.8% (95% CI 16.2-49.2) when the 6-12 week- old and 5-17 month- old groups are combined.

The only other malaria vaccination approach that has demonstrated repeatable partial efficacy in humans involves the use of virally vectored vaccines containing a recombinant genetic insert, encoding the antigen/s against which the immune response is targeted (Ewer et al, submitted, [67,68]). The candidate malaria vaccines ChAd63 ME-TRAP and MVA ME-TRAP consist of inactivated viral vectors (ChAd63 and MVA) containing the recombinant DNA insert, ME-TRAP. Vaccination induces immune responses against the pre-erythrocytic-stage *P. falciparum* antigenic epitopes encoded by ME-TRAP. Heterologous prime-boost vaccination with ChAd63 ME-TRAP prime, followed eight weeks later by MVA ME-TRAP



 © University of Oxford, 2013

boost, has shown durable partial efficacy against *P. falciparum* infection in a UK Adult Phase IIa sporozoite challenge study. Efficacy testing of this promising vaccination strategy in adults living in malaria-endemic areas began in Kenya in March 2012, and is planned to commence in Senegal in June 2012.

## 2.2 Investigational Products

### *Description of ChAd63 ME-TRAP and MVA ME-TRAP*

*The Modified Vaccinia virus Ankara (MVA) vector*

MVA is an attractive candidate orthopox vaccine vector for safety and immunogenicity reasons. The successful worldwide eradication of smallpox using vaccination with vaccinia virus highlighted vaccinia as a candidate carrier. Although millions of humans have been vaccinated with conventional replication-competent vaccinia virus, its small but definite risk to both researchers and future patients led to the development of several attenuated strains of vaccinia during smallpox eradication and more recently. In particular the host-range restricted MVA proved to be extremely attenuated compared to other vaccinia viruses.

MVA was originally derived from the vaccinia strain Ankara by over 500 serial passages in primary chicken embryo fibroblasts (CEF cells). MVA has six major genomic deletions compared to the parental Ankara genome and is severely compromised in its ability to replicate in mammalian cells. No replication has been documented in non-transformed mammalian cells. The viral genome has been proven to be stable through a large series of passages in chicken embryo fibroblasts[38]. MVA also showed no cytopathic effect or plaque formation in cells of human origin. In irradiated mice, MVA did not elicit any morbidity or lethality even when administered at high doses intra-cerebrally, indicating its safety even in immuno-compromised animals[38].

Apart from studies in mice, rabbits and elephants[39], MVA has been shown to be safe in humans[40]. From 1972 until 1980 (the end of compulsory smallpox vaccination) MVA was licensed in Germany[39] and was included in the official immunisation schedule[41]. In a large field study carried out in Germany in the late seventies, over 120,000 previously unvaccinated individuals were vaccinated with MVA (0.2 mL) administered either intra-dermally or subcutaneously. The study population included high-risk groups such as people suffering from allergies, elderly people and alcoholics. Given intradermally, a red nodule of up to 4 mm in diameter was observed at the injection site at day 4 or 5. Only a small proportion showed any systemic side effects such as fever > 38.5°C [38]. MVA proved to be non-contagious and avirulent. Viral replication is blocked late during infection of cells but importantly viral and recombinant protein synthesis is unimpaired even during this abortive infection. Replication-deficient recombinant MVA has been viewed as an exceptionally safe viral vector. When tested in animal model studies, recombinant MVAs have been shown to be avirulent, yet protectively immunogenic as vaccines against viral diseases and cancer[38]. Recent studies in macaques severely immuno-suppressed by SIV infection have further supported the view that MVA should be safe in immuno-compromised humans[42].

 © University of Oxford, 2013

*The ChAd63 vector*

Human adenoviruses (AdHu) are attractive viral vectors as they possess a genetically stable virion so that inserts of foreign genes are not deleted. Also, adenoviruses can infect large numbers of cells and the transferred information remains epichromosomal, thus avoiding any potential for insertional mutagenesis. Replication defective adenovirus can be engineered by deletion of genes from the E1 locus, which is required for viral replication, and these viruses can be propagated easily with good yields in cell lines expressing E1 from AdHu5 such as human embryonic kidney cells 293 (HEK 293)[44]. Previous mass vaccination campaigns using orally administered live human adenovirus serotype 4 and 7 in large numbers of US military personnel have shown good safety and efficacy data[45].

A limiting factor to the usage of human adenovirus as a vector is the level of anti-vector immunity present in humans where adenovirus is a ubiquitous infection. Estimates suggest that depending on the geographical region between 45–80% of adults carry AdHu5-neutralising antibodies[46]. Immunisation with AdHu vectors in animal models in the presence of pre-exposure to human adenoviruses attenuates responses to the vaccine probably due to the removal of virus particles by pre-existing antibodies[47-49]. Phase I trials of a multiclade HIV-1 vaccine delivered by a replication defective AdHu5 had to exclude volunteers with pre-existing antibodies to AdHu5 at titres greater than 1:12 [50]. In recent Phase I placebo controlled human trials of a modified AdHu5 HIV vaccine there were no safety concerns amongst vaccinated volunteers with pre-existing high titre anti-AdHu5 antibodies, indeed less reactogenicity was seen amongst those with high-titre antibodies[51]. Using AdHu5 in a prime boost strategy for HIV-1 gag homologous boosting did not improve the peak post prime levels of gag specific lymphocytes, probably due to anti-vector immunity[52].

The prevalence of immunity to human adenovirus prompted the consideration of simian adenoviruses as vectors. They exhibit hexon structures homologous to that of human adenoviruses[53]. Indeed, the chimpanzee adenovirus ChAd63's hexons are most similar in sequence to the hexons of AdHu4 previously used by the US military in mass vaccination campaigns where over 2 million adults received tablets of serially passaged adenovirus with good safety and efficacy data (Personal Communication Col. John D. Grabenstein)[54]. In chimpanzee adenoviruses the E1 locus can be deleted to render viruses replication deficient and allow transcomplementation on an E1 AdHu5 complementing cell line[55]. An additional attractive observation is that the lack of sequence homology between AdHu5 and simian adenoviruses at the E1 flanking sequence prevents homologous recombination and production of replication competent virus[56].

Simian adenoviruses are not known to cause pathological illness in humans and the prevalence of antibodies to chimpanzee origin adenoviruses is less than 5% in humans residing in the US[57]. In Equatorial Africa (the natural habitat for chimpanzees), prevalence is higher but still below that to anti AdHu5 immunity. In a recent study in Kenya, 23% of children aged 1-6 years had high-titre neutralising antibodies to AdHu5, whilst only 4% had high-titre neutralising antibodies to ChAd63 (also known as AdCh63). Immunity to both vectors was age-dependent[58]. A recent survey of 100 infants and children living in Banfora, Burkina Faso, has shown a 3% seroprevalence of neutralising antibodies to ChAd63 (MVVC,

 © University of Oxford, 2013

unpublished data). Early murine work using chimpanzee adenovirus 68 (AdCh68) expressing *gag* of HIV-1 showed that in comparison to AdHu5 and poxvirus, AdCh68 was as effective at generating a transgene product specific CD8+ T cell response with approximately 20% of all splenic CD8+ being gag specific[59]. In the same study, pre-exposure to AdHu5 abolished any protection offered by immunisation with AdHu5 but only slightly reduced that elicited by AdCh68, suggesting pre-exposure to human adenoviruses should not reduce the potency of the immune response generated to simian vectored vaccines.

There is no available or validated *in vitro* cell co-culture method to examine co-infection with human and simian adenovirus vectors as the latter are non-replicating. Due to a lack of any sequence homology between the replication-deficient ChAd63 and MVA vectors, complementation of MVA by ChAd63 does not occur. Pre-clinical biodistribution studies have demonstrated no viable persistence of the ChAd63 vector 24 hours post intramuscular administration. Therefore, residual priming ChAd63 vector is very unlikely to be present at the time of administration of a MVA boost, 8 weeks later.

*The ME-TRAP insert*

ME-TRAP is recombinant 2398 base-pair DNA insert which encodes for a single polypeptide of 789 amino acids[23,30] containing multiple epitopes (ME) and the *P. falciparum* pre-erythrocytic thrombospondin-related adhesion protein (TRAP). ME is a string of 20 epitopes, mainly CD8 T cell epitopes from *P. falciparum* pre-erythrocytic antigens. The individual CTL epitopes which constitute the 'multiple epitope' part of ME-TRAP are recognised by a number of common human HLA types, represent a variety (six) of potentially protective target antigens and are included to help ensure an immune response to the vaccine in the majority of the population vaccinated[72]. The ME string is fused to the entire sequence of the T9/96 strain of *P. falciparum* TRAP. TRAP is a well characterized and abundant pre-erythrocytic stage *P. falciparum* antigen and has a protective homologue in rodents[31].

## 2.3 Overview of ChAd63 ME-TRAP / MVA ME-TRAP Heterologous Prime-Boost Immunisation

***T cell responses to preerythrocytic malaria antigens***

Malaria immunity is complex, however T cell responses provide protection against malaria in animal models[10], in the field[11-13], following irradiated sporozoite inoculation[14-16] and following vaccination. Immunisation of mice with irradiated sporozoites of murine *Plasmodium* provides protection against later challenge with murine malaria sporozoites[10]. This protective immunity can be transferred to non immune mice by transferring the CD8+ T lymphocyte clones specific to pre-erythrocytic malaria surface antigens, the circumsporozoite protein (CS), or thrombospondin related adhesion protein (TRAP) that were induced by irradiated sporozoites[14,15]. In immune mice, depletion of CD8+ T cells renders them susceptible to further infection with *P. berghei* [17]. Incubation of infected hepatocytes with unfractionated spleen cells from immune animals resulted in elimination of infected hepatocytes[18]. This has been shown to be mediated by T lymphocyte recognition of a circumsporozoite protein derived peptide on infected mouse hepatocytes that provokes

 © University of Oxford, 2013

lysis of the infected cell and parasite death[19]. A recent study has confirmed the protective effect of repeated human inoculation with irradiated sporozoites against experimental *P. falciparum* challenge[16].

The class 1 Human Leucocyte Antigen (HLA) HLA B53, present in West African populations, has been associated with protection against severe forms of malaria in West African children. HLA B53 restricted cytotoxic T lymphocytes (CD8+) recognise a conserved peptide from liver-stage-specific antigen 1 (LSA-1) [11,12]. T cell memory responses, quantified by cultured ELISPOT to TRAP antigens, were associated with a reduced risk of clinical malaria in Kenya[13]. Conversely, *P. falciparum* infection has a deleterious effect on T cell responses, possibly increasing the risk of further malaria episodes[20].

This evidence has prompted the development of malaria vaccine strategies which induce T cell responses against antigens expressed by the malaria parasite during the pre-erythrocytic stage of its lifecycle. The TRAP antigen is the leading target for such strategies at Oxford[21]. The targeted mechanism of protection against malaria is the induction of a TRAP-specific cellular immune response that eliminates infected liver cells, preventing parasites in the liver reaching the blood stream, where clinical illness is initiated and where continuation of the parasite's lifecycle occurs. Vectored vaccines containing the ME-TRAP genetic insert are used to generate T cell responses against TRAP. In the heterologous prime-boost strategy, two different vectored vaccines, both containing ME-TRAP, are given in sequence. This achieves an expansion of T cells reactive to TRAP, rather than to the vectors used.

***Early vector platforms for heterologous prime-boost vectored vaccination strategies targeting TRAP***

Heterologous prime-boost vaccination with DNA containing ME-TRAP (DNA ME-TRAP) followed by recombinant MVA containing ME-TRAP (MVA ME-TRAP) led to significant reductions of an estimated 80% in parasite burden in the liver on human challenge with malaria infection[22-24]. In one study[24], one of eight vaccinees was sterilely protected and in another[23], there was a significant delay in the time to patency.

Following murine work which showed heterologous prime boost with FP9 and MVA was more immunogenic and protective than the DNA-MVA strategy, clinical trials using this approach were carried out in Oxford. Vaccination with FP9 ME-TRAP followed by MVA ME-TRAP led to an estimated 90% reduction in parasite burden in the liver, with two of sixteen vaccinees protected against malaria challenge[22,25]. Following these promising findings, studies were undertaken in adults and then children in Kilifi[26,27]. Immunogenicity was lower than expected[20], and efficacy was not seen in a study of 400 children in Kilifi district [28].

T cell responses are a correlate of protection induced by these vaccination strategies, as measured by delay in time to patency or reduction in parasite burden in the liver on malaria challenge[25,29]. Further development of T cell inducing vaccination in Oxford has therefore examined more immunogenic vectors in order to attain greater efficacy of the heterologous prime-boost strategy. This has led to the development of malaria vaccination strategies



 © University of Oxford, 2013

using adenoviral and MVA vectors in heterologous prime-boost vaccination strategies targeting TRAP.

Clinical trials with prime-boost vaccination using both the adenoviral vectored vaccine, ChAd63 ME-TRAP, and MVA ME-TRAP, commenced in 2007, although MVA ME-TRAP was first used in 1999, and have shown the strongest T cell immunogenicity to date, as well as efficacy in Phase IIa evaluation. A brief overview of the progress, to date, of clinical trials evaluation of ChAd63 ME-TRAP / MVA ME-TRAP prime-boost immunisation is provided below. Detailed findings of preclinical and clinical evaluation of ChAd63 ME-TRAP and MVA ME-TRAP (including ChAd63 ME-TRAP / MVA ME-TRAP prime-boost immunisation) are provided in the Investigator Brochures for these IMPs (ChAd63 ME-TRAP was formerly designated as “AdCh63 ME-TRAP”).

***Overview of Clinical Trials Evaluating Heterologous Prime Boost Immunisation with ChAd63 ME-TRAP followed by MVA ME-TRAP***

Phase I clinical testing of the safety and immunogenicity of heterologous prime boost immunisation with ChAd63 ME-TRAP followed eight weeks later by MVA ME-TRAP began in adults in the UK in October 2007 (VAC033 clinical trial[73]). Fifty four healthy volunteers received ChAd63 ME-TRAP alone, or followed by MVA ME-TRAP. ChAd63 ME-TRAP - MVA ME-TRAP prime-boost immunisation showed excellent safety and potent T cell immunogenicity. Vaccination was more potently immunogenic than earlier vector platforms delivering the ME-TRAP insert. Dose ranging evaluation indicated that the preferred doses for adults, balancing reactogenicity and immunogenicity, were 5 x $10^{10}$ vp for ChAd63 ME-TRAP, and 2 x $10^{8}$ vp for MVA ME-TRAP.

Adult phase I/IIa testing of safety, immunogenicity and efficacy commenced in the UK in February 2009 (MAL034 clinical trial). Efficacy was evaluated in healthy adult volunteers in the UK using sporozoite challenge by *P. falciparum*-infected mosquitoes. ChAd63 ME-TRAP / MVA ME-TRAP prime boost immunisation provided durable partial efficacy against experimental malaria infection, with 3 of 14 challengees showing sterile protection (21%) and a further 5 showing partial efficacy corresponding to a >90% reduction in liver parasite burden, amounting to 8 / 14 (57%) vaccinees showing significant vaccine efficacy in this challenge model (Ewer et al submitted for publication).

Phase I clinical testing of ChAd63 ME-TRAP / MVA ME-TRAP heterologous prime-boost immunisation in populations living in malaria-endemic areas began in June 2010 in Kenya (VAC040 clinical trial) and The Gambia (VAC041 clinical trial, adult arm). A total of 46 healthy men received prime-boost immunisation at the above preferred adult doses. The vaccination strategy showed an excellent safety profile, with vaccinations being well tolerated and adverse events generally mild in grade. The potent T cell immunogenicity seen in UK volunteers was maintained in malaria-endemic populations, with no significant reduction in the malaria-endemic setting compared to the UK. On comparison of the intradermal and intramuscular routes of administration of MVA ME-TRAP, the intramuscular route of administration showed a nonsignificant trend to reduced immunogenicity, and much improved local reactogenicity. ChAd63 ME-TRAP has been evaluated clinically in Africa only by the intramuscular route.



 © University of Oxford, 2013

Age de-escalation of the assessment of safety and immunogenicity in malaria-endemic areas commenced in 2-6 year old Gambian children in January 2011 (VAC041 paediatric arm). 24 children received ChAd63 ME-TRAP, of whom 22 received MVA ME-TRAP eight weeks later. ChAd63 ME-TRAP was evaluated at 1 x $10^{10}$ vp and 5 x $10^{10}$ vp, and MVA ME-TRAP was evaluated at 1 x $10^8$ pfu and 2 x $10^8$ pfu, all by the intramuscular route. All vaccinations were well tolerated, with adverse events being generally mild in intensity. The higher dose of MVA ME-TRAP had greater reactogenicity, without clearly improved immunogenicity, and therefore the lower dose was chosen for clinical evaluation in younger age groups. In VAC042, 24 Gambian infants aged 5-12 months have received ChAd63 ME-TRAP / MVA ME-TRAP prime-boost immunisation at doses of 1 x $10^{10}$ vp and 5 x $10^{10}$ vp for ChAd63 ME-TRAP and 1 x $10^8$ pfu for MVA ME-TRAP. All vaccinations have been well tolerated, with generally mild adverse events. ChAd63 ME-TRAP / MVA ME-TRAP prime-boost immunisation shows potent T cell immunogenicity in 2-6 year old children and 5-12 month old infants living in a malaria-endemic area.

Other ongoing and planned clinical evaluation of ChAd63 ME-TRAP / MVA ME-TRAP includes the following:

- A UK Phase I adult clinical trial (VAC043) of the safety and immunogenicity of novel schedules of administration of ChAd63 ME-TRAP and MVA ME-TRAP. Each of seven schedules incorporates four administrations of vaccine, with different sequences and time intervals. Interim findings indicate preserved safety, tolerability and immunogenicity with several alternative schedules of administration, and the ability to re-boost immune responses with repeated vaccinations.
- Phase IIb evaluation of the efficacy of ChAd63 ME-TRAP / MVA ME-TRAP heterologous prime-boost immunisation against *P. falciparum* malaria in adults living in malaria endemic populations (VAC046 and VAC047). The VAC046 study, which commenced enrolment in March 2012, will measure efficacy against natural *P. falciparum* infection in the 2012 peak malaria season in Kenya. Plans are underway to commence VAC047 in 2012, which will assess efficacy in a similar fashion, in Senegal

***Overview of the Safety of ChAd63 ME-TRAP***

Full details of the safety evaluation of ChAd63 ME-TRAP are provided in the Investigator's Brochure. In summary, ChAd63 ME-TRAP has been administered to 309 healthy adults, children and infants in clinical studies in the UK and Africa, generally as the priming vaccination prior to MVA ME-TRAP. All recipients of ChAd63 ME-TRAP have been healthy volunteers participating in clinical trials which have evaluated the safety of vaccination. ChAd63 ME-TRAP has an excellent safety record. Vaccine related adverse events are generally mild in grade, and resolve completely. There have been no vaccine related serious adverse events or SUSARs. Vaccination may provoke a mild local inflammatory reaction. This may include pain, warmth, redness and swelling. Moderate or severe local reactions are unlikely. The intramuscular route is associated with reduced local reactogenicity. Common systemic adverse events resulting from ChAd63 ME-TRAP include headache, feverishness, fatigue and malaise. Volunteers may report a transient

 © University of Oxford, 2013

mild 'flu-like illness within 24-48 hours of vaccination which resolves rapidly. Moderate or severe adverse events are unlikely.

***Overview of the Safety of MVA ME-TRAP***

Full details of the safety evaluation of MVA ME-TRAP are provided in the Investigator's Brochure. In summary, MVA ME-TRAP has been administered in phase I and II trials in the UK, The Gambia and Kenya and has been shown to be safe and immunogenic[30, 32, 23]. 772 adults, children and infants in the UK and Africa have received MVA ME-TRAP. Volunteers have received one to three doses of MVA ME-TRAP intra-dermally or intramuscularly at doses of $3 \times 10^7$ – $2 \times 10^8$ pfu alone or as part of heterologous prime boost regimes incorporating DNA ME-TRAP, FP9 ME-TRAP and most recently ChAd63 ME-TRAP. Vaccination with MVA ME-TRAP has been well tolerated by all vaccinees to date and the majority of adverse events have been mild in nature. The frequency and intensity of adverse events increases with the dose of MVA ME-TRAP. There have been no vaccine-related SAEs or SUSARs. With intramuscular administration of MVA ME-TRAP, the majority of volunteers experience one or more local adverse event/s such as local pain, warmth and swelling. Local adverse events are generally mild or moderate in grade. Systemic side effects can occur with MVA ME-TRAP and appear to be dose related with symptoms occurring at a higher frequency and greater intensity in volunteers receiving higher doses. Most commonly reported are the sensation of feverishness, malaise, arthralgia, myalgia, headache and nausea or vomiting. Systemic adverse events are generally mild in grade.

## 3. RATIONALE

**Vaccine Development Strategy**



 © University of Oxford, 2013

The main population that would benefit from an effective *P. falciparum* vaccine would be infants and children living in malaria-endemic areas in Africa. The malaria vaccine strategy most advanced in clinical development, RTS,S, shows partial efficacy[69,70], of about a 35% reduction in malaria episodes in the year following vaccination, and therefore efforts to reduce the burden of malaria should continue to develop other candidate vaccination strategies which could give rise to more efficacious approaches, or which could be combined with RTS,S or a biosimilar CS-based vaccine to provide a more effective vaccine.

ChAd63 ME-TRAP / MVA ME-TRAP heterologous prime-boost immunisation is a highly promising candidate malaria vaccination strategy. It shows durable partial efficacy in malaria challenge studies in the UK, and a CD8+ T cell correlate of efficacy has been identified (Ewer et. al., submitted; IB for MVA ME-TRAP) which may help predict the potential efficacy of the strategy based on immunogenicity results of clinical studies. The vaccination strategy shows potent immunogenicity in malaria-endemic populations, including infants and children, and has an excellent track record of safety. Combination of this approach with RTS,S may also achieve improved efficacy. Indeed, there is pre-clinical evidence that there might be more than additive efficacy of an anti-sporozoite vaccine, such as RTS,S, and an anti-liver-stage vaccine such as ME-TRAP in vectors. Clinical evaluation indicates that there may be flexibility to modify the ChAd63 ME-TRAP / MVA ME-TRAP vaccination regimen to be compatible with EPI programmes if necessary for field-applicability in infants and children, and to boost immune responses over a longer period of time with repeat vaccination. Preclinical testing indicates that there may be scope to use adjuvants to improve the performance of this vaccination strategy. For all of these reasons, ChAd63 ME-TRAP / MVA ME-TRAP prime-boost immunisation is a promising candidate malaria vaccination strategy which warrants further clinical development with a view to developing a highly effective multi-stage *P. falciparum* vaccine.

Building on these findings, we seek to evaluate the efficacy of this vaccination strategy in infants and children living in malaria-endemic areas. Undertaking this study in Burkina Faso is justified by the high incidence of malaria mortality and morbidity here, and in the rest of sub-Saharan Africa. There is pressing need for an effective vaccination. While insecticide-treated bed nets, vector control measures and new cheap anti-malarial drug development are all important aspects of malaria control, a co-existing vaccine development programme is essential. The vaccination strategy proposed here has the potential to protect against naturally acquired infection in infants and children in Burkina Faso. A recent serosurvey of 6 month to 3 year olds living in Banfora showed a very low prevalence (3%) of neutralising antibodies to the ChAd63 vaccine vector (MVVC, unpublished data).

Dependent upon findings of the proposed trial, future clinical development of ChAd63 ME-TRAP / MVA ME-TRAP heterologous prime-boost immunisation could include

- assessment of the compatibility of ChAd63 ME-TRAP / MVA ME-TRAP heterologous prime boost vaccination with the existing EPI vaccination programmes for African infants.
- field testing of the combination of ChAd63 ME-TRAP / MVA ME-TRAP with RTS,S or a biosimilar CS-based vaccine, or other candidate malaria vaccines
- Phase III clinical testing of ChAd63 ME-TRAP / MVA ME-TRAP as used in this trial

 © University of Oxford, 2013

**Vaccination Regimen for the proposed trial**

The vaccination schedule to be evaluated, ChAd63 ME-TRAP followed eight weeks later by MVA ME-TRAP, is that which has undergone extensive clinical evaluation to date, and has shown durable partial efficacy in a UK Phase IIa sporozoite challenge trial (MAL034).

ChAd63 ME-TRAP and MVA ME-TRAP will be administered intramuscularly at doses of 5 x $10^{10}$ vp and 1 x $10^{8}$ pfu, respectively, both by the intramuscular route. These are the doses and route that have shown satisfactory safety and immunogenicity in prime-boost sequence in 5-12 month old Gambian infants (VAC042), as well as other populations. The intramuscular route is practicable for infants and children, and demonstration of efficacy with the intramuscular route would also facilitate potential future combination with RTS,S, which is also given by the intramuscular route.

There is additional data from another vaccination regimen to support the safety of the dose selected for MVA ME-TRAP. Satisfactory safety was seen when MVA ME-TRAP 1.5 x $10^{8}$ pfu was administered intradermally to 190 Kenyan children aged 1 to 6 years following two sequential FP9 ME-TRAP vaccinations[28.] The vaccines were well tolerated and there were no laboratory abnormalities attributed to vaccination.

## 4. OBJECTIVES

The objective of the Lead in safety evaluation is to assess the safety of ChAd63 ME-TRAP / MVA ME-TRAP prime-boost immunisation in a cohort of 5-17 month old Burkinabe infants and children.

The objectives of the Phase 2b trial are as follows:



 © University of Oxford, 2013

*<u>Primary Objective</u>*

To assess the protective efficacy against clinical malaria of ChAd63 ME-TRAP / MVA ME-TRAP prime-boost immunisation, in 5-17 month old infants and children living in a malaria-endemic area, for 6 months after the last vaccination

*<u>Secondary Objectives</u>*

*Duration of Protective efficacy against clinical malaria*
To assess the protective efficacy against clinical malaria of ChAd63 ME-TRAP / MVA ME-TRAP prime-boost immunisation, in 5-17 month old infants and children living in a malaria-endemic area, for 12 and 24* months after the last vaccination.

*Efficacy against asymptomatic P. falciparum infection*
To assess the protective efficacy against asymptomatic *P. falciparum* infection of ChAd63 ME-TRAP / MVA ME-TRAP prime-boost immunisation, in 5-17 month old infants and children living in a malaria-endemic area, 6, 12 and 24* months after the last vaccination

*Efficacy against secondary case definitions of clinical malaria*[+]
To assess the protective efficacy against secondary case definitions of clinical malaria of ChAd63 ME-TRAP / MVA ME-TRAP prime-boost immunisation, in 5-17 month old infants and children living in a malaria-endemic area, for 6, 12 and 24* months after the last vaccination

*Safety Objective*
To assess the safety and reactogenicity of ChAd63 ME-TRAP / MVA ME-TRAP heterologous prime-boost immunisation, in 5-17 month old infants and children living in a malaria-endemic area, for 6, 12 and 24 months after the last vaccination.

*Immunogenicity Objectives*
- To assess the immunogenicity of ChAd63 ME-TRAP / MVA ME- TRAP heterologous prime-boost immunisation, in 5-17 month old infants and children living in a malaria-endemic area.
- To explore the immunologic correlates of protective efficacy of ChAd63 ME-TRAP / MVA ME-TRAP prime-boost immunisation, in 5-17 month old infants and children living in a malaria-endemic area.

*<u>Exploratory Objective</u>*

*Efficacy against incident cases of severe malaria*
To assess the protective efficacy against severe malaria of ChAd63 ME-TRAP / MVA ME-TRAP prime-boost immunisation, in 5-17 month old infants and children living in a malaria-endemic area, for 6, 12 and 24* months after the last vaccination.

 © University of Oxford, 2013

# 5. DESCRIPTION AND JUSTIFICATION OF STUDY DESIGN

**Overview**

A double blind randomised controlled trial, with a lead-in safety evaluation, is proposed to evaluate the efficacy of ChAd63 ME-TRAP / MVA ME-TRAP heterologous prime-boost immunisation in healthy 5-17 month old children and infants in a malaria endemic area.

**Lead-in Safety Evaluation followed by Phase 2b trial**

Confirmation of satisfactory safety of the vaccines in a small sample of the local population of Burkinabe infants and children is desirable before vaccinating the larger number of participants in the Phase 2b trial. Therefore there will be interim assessments of vaccine safety in 30 participants (Group A), with DSMB approvals required to administer vaccines to Group B and C infants. This is detailed in Section 8.


© University of Oxford, 2013

For the Phase 2b trial, participants will be randomised 1:1 to receive vaccination with the IMPs (ChAd63 ME-TRAP and MVA ME-TRAP; Group B) or control vaccination with Rabies Vaccine (Group C). Participants and investigators will be blinded to whether the participant is in Group B or C. Efficacy of vaccination will be assessed by comparing the development of malaria between Group B versus Group C participants.

**Vaccinations**

There are three study vaccines: the two IMPs, ChAd63 ME-TRAP and MVA ME-TRAP; and Rabies Vaccine. Group A (Lead-in safety Evaluation) and Group B (active vaccine group for Phase 2b trial) participants will receive ChAd63 ME-TRAP 5 x $10^{10}$ vp followed eight weeks later by MVA ME-TRAP 1 x $10^{8}$ pfu, both intramuscularly. The same anterolateral thigh will be used for both vaccinations.

Group C (control group for Phase 2b trial) participants will receive two vaccinations with Rabies Vaccine, eight weeks apart, both given intramuscularly. The same anterolateral thigh will be used for both vaccinations. Rabies vaccinations should provide some protection against rabies[74,75], are anticipated to be well tolerated, and are anticipated to cause some local and systemic reactogenicity that will facilitate the blinding of investigators to whether the participant received rabies vaccination or ChAd63 ME-TRAP / MVA ME-TRAP.

There will be a minimum one week interval between administration of any study vaccine and any EPI vaccine. This is as a precaution to avoid interference between the immunogenicity of the vaccines, and also to facilitate assessment of study vaccine-related AEs, independent of EPI vaccine- related AEs. There is data from the TB012 study[61] supporting the non-interference of another MVA-vectored vaccine, MVA85A, with EPI vaccines. In this study in Gambian infants, even simultaneous administration of MVA85A (considered a boost vaccination after BCG prime) did not appear to interfere with the immunogenicity of the EPI vaccines. Antibody titres against each vaccine component in the DTwP-Hib and Hepatitis B regimen of the Gambian EPI were compared between subjects receiving MVA85A and EPI simultaneously, and subjects receiving EPI vaccines alone. The geometric mean or median antibody titre to each of the antigens was not significantly different between the groups. Further analysis showed no significant difference between the groups in the proportions of subjects that achieved levels above the protective thresholds. It was concluded that administration of MVA85A did not significantly affect the humoral response to the EPI vaccines.

All participants will be offered rabies vaccination by the end of the trial so that any benefit of reduced susceptibility to rabies through vaccination is not provided selectively to the control group.

**Endpoints for the Lead-in Safety Evaluation**

Description of local and systemic solicited and unsolicited adverse events considered possibly, probably or definitely related to vaccination with ChAd63 ME-TRAP and MVA ME-TRAP, and line listing of all SAEs.

 © University of Oxford, 2013

Solicited injection site adverse events are pain/limitation of limb movement, swelling and redness/discoloration. Solicited systemic adverse events are fever, loss of appetite, irritability, and drowsiness.

**Endpoints for the Phase 2b Trial**

***Safety endpoints***

SAEs occurring from first vaccination until the end of the study
Local and systemic solicited and unsolicited adverse events, considered possibly, probably, or definitely related to vaccination, occurring from first vaccination until 1 month post second vaccination (study day 93).

Solicited injection site adverse events are pain/limitation of limb movement, swelling and redness/discoloration. Solicited systemic adverse events are fever, loss of appetite, irritability, and drowsiness.

***Immunogenicity endpoints***

Immune responses and their determinants. These may include:

- T cell enumeration and characterisation, using ELISPOT, and flow cytometry with intracellular cytokine staining
- Measurement of antibodies to TRAP and other malaria antigens, using ELISA
- Measurement of antivector immune responses
- Enumeration of antibody-secreting cells, using ELISPOT and flow cytometry
- Cytokine quantification in serum, using ELISA
- Measurement of antibody response to other EPI vaccines administered to the participants Evaluation of genetic determinants of clinical outcome and immune responses to both TRAP and other malarial antigens and EPI vaccines using HLA typing, DNA and RNA analysis, detection of Haemoglobin gene variants, and other methods
- Transcriptional profiling

Study of the associations between immune responses and efficacy of ChAd63 ME-TRAP / MVA ME-TRAP prime-boost vaccination

***Efficacy Endpoints***

*Primary case definition of clinical malaria episode:* presence of
-Axillary Temperature ≥37.5°C AND
-*P. falciparum* parasites density > 5000 asexuals forms/µL

*Secondary case definitions of clinical malaria episode*

 © University of Oxford, 2013

a) Presence of
   -Axillary Temperature ≥37.5°C and/ or History of fever within the last 24 hours; AND
   - *P. falciparum* parasites density > 0

b) Presence of
   -Axillary Temperature ≥37.5°C; AND
   - *P. falciparum* parasites density > 500 asexuals forms/µL

c) Presence of
   -Axillary Temperature ≥37.5°C; AND
   - *P. falciparum* parasites density > 20,000 asexuals forms/µL

Either definition a) or b) or c) is sufficient for a secondary definition diagnosis of clinical malaria

*Primary case definition of asymptomatic P. falciparum infection:* presence of
-Axillary Temperature < 37.5°C and absence of History of fever within the last 24 hours; AND
- *P. falciparum* parasites density > 0 asexual forms/µL

*Primary case definition of severe malaria*: Presence of
-*P. falciparum* parasites density > 5000 asexuals forms/µL; AND
-one of more of the following criteria of disease severity:

- Prostration
- Respiratory distress
- Blantyre coma score ≤ 2
- Seizures: 2 or more
- Hypoglycemia < 2.2 mmol/L
- Acidosis BE ≤-10.0 mmol/L
- Lactate ≥ 5.0 mmol/L
- Anemia < 5.0 g/dL; AND

-Without any of the following criteria of co morbidity

- Pneumonia (confirmed by X-ray)
- Meningitis (confirmed by CSF examination)
- Sepsis (with Positive blood culture)
- Gastroenteritis with dehydration

*Secondary case definitions of severe malaria*.

a) Presence of:
-*P. falciparum* parasites density > 5000; AND
-With one or more of the following criteria of disease severity:

   - Prostration
   - Respiratory distress
   - Blantyre coma score ≤ 2
   - Seizures 2 or more
   - Hypoglycemia < 2.2 mmol/L
   - Acidosis BE ≤-10.0 mmol/L

 © University of Oxford, 2013

- Lactate ≥ 5.0 mmol/L
- Anemia < 5.0 g/dL

b) Presence of:
-*P. falciparum* parasites density > 0; AND
-With one or more of the following criteria of disease severity:

- Prostration
- Respiratory distress
- Blantyre score ≤ 2
- Seizures 2 or more
- Hypoglycemia < 2.2 mmol/L
- Acidosis BE ≤-10.0 mmol/L
- Lactate ≥ 5.0 mmol/L
- Anemia < 5.0 g/dL; AND

-Without any of the following criteria of co morbidity

- Pneumonia (confirmed by X-ray)
- Meningitis (confirmed by CSF examination)
- Sepsis (Positive blood culture)
- Gastroenteritis with dehydration

Either definition a) or definition b) is sufficient for a secondary definition diagnosis of severe malaria

**Study site**

The study will take place at the Banfora trial site, which is located about 400 km from Ouagadougou, the capital city of Burkina Faso. The URC-B, the research unit is situated within the complex of the Regional hospital. The trial participants will be drawn from Banfora Health demographic system that covers a total population of 30,000 inhabitants. From recent surveys, the bed net coverage was 80%. There is no implementation of Indoor Residual Spray or IPT in infants or children in the area. To date there is no evidence of the decline in malaria incidence that has been recently reported from other parts of sub-Saharan Africa. In Burkina Faso, malaria is endemic. Transmission occurs throughout the year, with a peak during the rainy season (June to October). *P. falciparum* is responsible for more than 90% of all clinical malaria cases. The major vectors are *Anopheles gambiae, An. arabiensis and An. funestus*. Children under five years and pregnant women are the populations at highest risk. During the five months when transmission reaches a peak, individuals in these age-brackets may suffer multiple malaria episodes, with an annual malaria death toll reaching 15,000 people. In the country's hospitals, malaria is reportedly responsible for 30.7% of all hospitalization with a mortality rate of 23%.

**Sample size**

 © University of Oxford, 2013

For the Lead-in Safety Evaluation, a sample of size of 30 participants has been selected as the minimum number required to provide adequate safety data to enable an interim assessment of safety which could support progressing to the Phase 2b trial.

The primary endpoint of the Phase 2b trial is the time to first episode of malaria meeting the primary case definition of clinical malaria episode, over a period of 6 months. We will aim to time vaccinations so that this 6 month interval (study day 63 to 243) incorporates the malaria season, which runs from June to October.

From the results of a baseline site survey, it is anticipated that during the malaria season, the incidence of malaria meeting the primary case definition of clinical *P. falciparum* malaria episode will be 50% in 5-17 month old infants. Rabies vaccination should not provide any protection against malaria infection. Therefore, the incidence of episodes meeting the primary case definition of clinical *P. falciparum* malaria between Day 63 and Day 243 in Group C should be 50%.

An efficacy of vaccination of 30% is that considered suitable for the vaccine strategy to be included in a future multi-component high efficacy vaccine. With a total sample of 700 participants, the expected power to detect 30% vaccine efficacy, (i.e., a hazard ratio of 0.7, with the proportion of malaria in Group B by Day 243 equalling 38%) will be 88%. Due to the short duration of the study, we anticipate the rate of loss to follow-up or drop out will be minimal. However, even with 20% drop-out or loss to follow-up, the sample size still gives 80% power to detect 30% vaccine efficacy.

| Proportion who develop clinical *P. falciparum* malaria in Group C | Proportion who develop clinical *P. falciparum* malaria in Group B | Hazard ratio | Power |
|---|---|---|---|
| 50% | 38% | 0.7 | 88% |
| 40% | 30% | 0.7 | 79% |

Table 2: Power to assess 30% efficacy of vaccination in the Phase 2b trial

**Rationale for the use of passive surveillance in the trial site**

For the assessment of the efficacy objectives of the Phase 2b trial, occurrence of malaria will be ascertained through passive surveillance (detailed in Section 8). A pilot study was conducted at the study site to assess the incidence of malaria episodes using passive surveillance and active surveillance. In the active surveillance cohort, children were visited twice a week at home by the research team to detect clinical malaria episodes. In the passive surveillance cohort, the caregivers were encouraged to take their child to the local health facility where the research team was based at any time the child felt sick.

The incidence of clinical malaria was 0.09 episodes per child per month at risk (95% ci [0.08, 0.11]) in the active cohort compared to 0.09 episodes per child per month at risk (95% ci

 © University of Oxford, 2013

[0.07, 0.11] in the passive cohort. The passive cohort was therefore found to be the most cost-effective approach for use in future trials having clinical malaria as an efficacy endpoint.

**Blinding (Phase 2b trial)**

Double-blinding will be used to reduce bias in evaluating the study endpoints. Double-blinding in this context means that the vaccine recipient, their parent(s)/guardian(s), all investigators and the study team responsible for the evaluation of efficacy, safety and immunogenicity endpoints will all be unaware of the exact treatment, (IMPs or rabies vaccine) given to the participant. The only study staff aware of the vaccine assignment for IMP or rabies vaccine will be those responsible for the storage and preparation of vaccines; these staff will play no other role in the study. The vaccines will be different in terms of volume and colour. Therefore, the contents of the syringe will be masked with an opaque label to ensure that parent(s)/guardian(s) as well as nurse administering the vaccine are blinded.

To ensure treatment concealment, allocation to Group B vs C will be done using sequentially numbered opaque sealed envelopes which an independent statistician will prepare and seal, and then provide to the investigator. The independent statistician will not be part of the study team. The study pharmacist will only be allowed to open an envelope after ensuring that the infant / child before him has met all eligibility criteria and has been given a study ID number. For each infant / child, eligibility will have to be counter checked and signed by a second person before allocation of study ID number. All envelopes will be retained to be checked by the clinical monitor.

The local safety monitor who is independent from the study team will also be provided with the randomisation of Groups B and C. If deemed necessary for reasons such as safety, the Local Safety monitor will unblind the specific enrolled subject without revealing the study group to the investigators.

***Study duration and timeline***

Proposed timeline for the study:

| Date | Activity |
|---|---|
| October 2012 | Commencement of Recruitment |
| December 2012 | First vaccination of Group A participants |
| January- February 2013 | Second vaccination of Group A participants |
| March-April 2013 | First vaccination of Group B and C participants |
| May 2013 | Second vaccination of Group B and Group C participants |
| May/June 2013 – December 2013 | Collection of endpoints for the primary analysis of efficacy |
| To May 2015 | Efficacy*, safety, and immunogenicity follow-up |

 © University of Oxford, 2013

Depending on whether significant efficacy is seen, and the potential of the vaccines for licensure, we may consider extending the follow up further for pharmacovigilance purposes. In such a case, informed consent will be obtained from study participants.

* Depending on the outcome of the efficacy analysis of the 12 month data (to June 2014), this may be extended to 24 months following completion of vaccination.

**Risks and Benefits**

The risks of study participation are those relating to vaccination and blood sampling.

Participating infants will receive two vaccinations with licensed rabies vaccine eight weeks apart, or vaccination with ChAd63 ME-TRAP followed eight weeks later by MVA ME-TRAP. Rabies vaccination is expected to be generally well tolerated. It may cause local reactions at the injection site such as pain and swelling or induration, and less commonly there may be fever as a systemic reaction. These reactions should generally be mild and resolve completely.

ChAd63 ME-TRAP has shown an excellent safety profile in adults, children and infants. It may cause minor injection site reactions such as swelling, and systemic reactions such as fever, but these appear to be uncommon, generally mild in severity, and resolve completely within a few days. There have been a few cases of elevated ALT following vaccination with ChAd63 ME-TRAP; these have been isolated changes not associated with any sequelae or evidence of organ dysfunction; monitoring for such elevations will be done in this study, and should such elevations occur they would be expected to resolve completely.

MVA ME-TRAP has also shown an excellent safety profile in adults, children and infants, and this includes as a boosting vaccination following ChAd63 ME-TRAP. Adverse events with the $1 \times 10^8$ pfu dose selected for this study should be generally mild, and may include pain, swelling and skin redness/discoloration at the injection site, and fever. These would be expected to resolve completely.

As with any vaccine, serious allergic reactions including anaphylaxis may occur. Such problems are very rare events with any vaccine and have never occurred with AdCh63 ME-TRAP, or any other simian adenovirus-based vaccine, or MVA ME-TRAP. The incidence is unknown, but is estimated at one per $10^5$ to $10^6$ immunisations. Volunteers will be vaccinated in a clinical area where Advanced Life Support drugs and equipment are immediately available for the management of serious adverse reactions.

Blood collection may be associated with some discomfort and local bruising. The volume of blood collected for the research will not exceed 1ml/kg at any one time, and will not exceed 3-4 such blood samplings over eight weeks. These blood volumes are anticipated to be acceptable to parents/guardians and safe[79.80] for the infants deemed eligible to participate in this study.

 © University of Oxford, 2013

Participants will not benefit directly from vaccination with ChAd63 ME-TRAP and MVA ME-TRAP, but may be afforded some protection against rabies by the rabies vaccine[74,75]. Parents/guardians of participating infants will be counselled that they should not expect that study vaccination will provide any protection against malaria, and that participating in the study does not reduce the need for preventive measures against malaria.

## 6. Inclusion and Exclusion Criteria

The inclusion criteria will be used at Screening (see study procedures, Section 8) to identify participants eligible for the study, and will be checked prior to vaccination to confirm ongoing eligibility. Eligible infants will fulfil all of the inclusion criteria and none of the exclusion criteria.

**Inclusion Criteria**

1. Healthy infant/child aged 5-17 months at the time of first study vaccination
2. Informed consent of parent/guardian
3. Infant / child and parent/guardian resident in the study area villages and anticipated to be available for vaccination and follow-up

**Exclusion Criteria**

Any of the following constitutes an exclusion criterion:

 © University of Oxford, 2013

- Clinically significant skin disorder (psoriasis, contact dermatitis etc.), immunodeficiency, cardiovascular disease, respiratory disease, endocrine disorder, liver disease, renal disease, gastrointestinal disease, neurological illness.
- Weight-for-age *Z* score of less than –3 or other clinical signs of malnutrition
- History of allergic reaction, significant IgE-mediated event, or anaphylaxis to immunisation
- History of allergic disease or reactions likely to be exacerbated by any component of the vaccines, e.g. egg products, Kathon, neomycin, beta-propiolactone.
- Haemoglobin less than 8.0 g/dL, where judged to be clinically significant in the opinion of the investigator
- Serum Creatinine concentration greater than 70 µmol/L, where judged to be clinically significant in the opinion of the investigator
- Serum ALT concentration greater than 45 U/L, where judged to be clinically significant in the opinion of the investigator
- Blood transfusion within one month of enrolment
- Previous vaccination with experimental malaria vaccines.
- Administration of any other vaccine or immunoglobulin less than one week before vaccination with any study vaccine.
- Current participation in another clinical trial, or within 12 weeks of this study.
- Any other finding which in the opinion of the investigators would increase the risk of an adverse outcome from participation in the trial or result in incomplete or poor quality data
- Known maternal HIV infection (No testing will be done by the study team)
- Immunosuppressive therapy (steroids, immune modulators or immune suppressors) within 3 months prior recruitment. (For corticosteroids, this will mean prednisone, or equivalent, ≥ 0.5 mg/kg/day. Inhaled and topical steroids are allowed.)

**Withdrawal criteria**

Every reasonable effort will be made to maintain protocol compliance and participation in the study. If a subject is withdrawn from the study, the reason will be recorded. If withdrawal is the result of a Serious Adverse Event, the investigator will offer to arrange for appropriate management of the problem and the Ethical Committee will be informed in a timely manner. The extent of follow up will be determined by the investigator but will be at least for the whole study period. Subjects withdrawn prematurely for any reason will not be re-entered in to the trial, although they may be requested to return to the clinic for safety evaluation, and they will be included in study evaluations. A complete safety evaluation will be made for any subject who terminates from the study prematurely. If possible within the study period, volunteers will be replaced.

Subjects may be withdrawn from the study:

- By withdrawal of parental consent
- On the decision of the Investigator
- On the advice of the DSMB

The Investigator may withdraw the subject for any of the following reasons:

 © University of Oxford, 2013

- Any adverse event which results in inability to comply with study procedures
- Ineligibility either arising during the study or retrospectively (having been overlooked at screening)
- Significant protocol deviation
- Loss to follow up (applies to a subject who consistently does not return for protocol study visits, is not reachable by telephone or other means of communication and/or is not able to be located)

# 7. Investigational Medicinal Products

There are three study vaccines: the two investigational medicinal products, ChAd63 ME-TRAP and MVA ME-TRAP; and Rabies Vaccine.

**Formulation and Dose of Investigational Medicinal Products**

ChAd63 ME-TRAP

ChAd63 ME-TRAP is manufactured under Good Manufacturing Practice conditions by the Clinical Biomanufacturing Facility (CBF), Churchill Hospital, Oxford. ChAd63 ME-TRAP is supplied as a sterile 0.5-1.0 ml liquid in 2.0ml glass vials. The dose of ChAd63 ME-TRAP used in this study is 5 x $10^{10}$ vp.

MVA ME-TRAP

MVA ME-TRAP is manufactured under Good Manufacturing Practice conditions by IDT Biologika GmbH (IDT), Germany. MVA ME-TRAP is supplied as a sterile 0.55ml liquid in 2.0 mL clear glass injection vials. The dose of MVA ME-TRAP to be used in this study is 1 × $10^8$ pfu.

 © University of Oxford, 2013

**Storage, dispensing and handling of Investigational Medicinal Products**

Accountability, storage, shipment and handling of IMPs will be in accordance with relevant SOPs and forms. All movements of IMPs will be documented in vaccine accountability logs according to local site SOPs.

ChAd63 ME-TRAP and MVA ME-TRAP are stored between –70$^0$C and –90$^0$C in a locked freezer at the University of Oxford, Churchill Hospital. The vaccines will be shipped from Oxford on dry ice, and then stored in a –70$^0$C freezer at the CNRFP until required. The vaccines will be transported to Banfora trials Centre and stored locally in -80°C freezer.

**Administration of study vaccines**

For Group A, vaccination on Day 0 is with ChAd63 ME-TRAP, and vaccination on Day 56 is with MVA ME-TRAP. The staff administering the vaccine may both prepare the vaccine in the administration syringe and administer it.

For the Phase 2 Trial, vaccination on Day 0 is with ChAd63 ME-TRAP (Group B) or Rabies Vaccine (Group C), and vaccination on Day 56 is with MVA ME-TRAP (Group B) or Rabies Vaccine (Group C). The staff responsible for the storage and preparation of study vaccines will be aware of Group allocation, and will prepare the vaccine in the administration syringe accordingly. They will mask the contents of the administration syringe with an opaque label to ensure that the parent(s)/guardian(s), the staff administering the vaccine, and all other staff, remain blinded to the Group allocation of the study participant. The staff responsible for the storage and preparation of the vaccines will play no other role in the study, so that blinding is maintained.

Vaccinations will be administered intramuscularly in to the anterolateral thigh. The same side will be used for both vaccinations.

ChAd63 ME-TRAP and MVA ME-TRAP are genetically modified organisms. In order to minimise dissemination of the recombinant vectored vaccine viruses into the environment, the inoculation site for all vaccinations will be covered with a dressing after immunisation. This should absorb any virus that may leak out through the needle track, and will be removed from the injection site after 30 minutes. Vaccine administrators will follow precautions for the safe handling of GMOs (including the use of eye protection and gloves).

Each volunteer will be monitored for one hour (or longer if necessary) after each vaccination. Resuscitation (including intubation) equipment and medication will be available in the clinic site and a clinician trained in resuscitation will be present at all times during this immunisation time period.

**Contraindications to vaccine administration**

The following constitute contraindications to administration of vaccine at that point in time. If any one of these occurs at the time scheduled for vaccination, the subject may be

 © University of Oxford, 2013

vaccinated at a later date or withdrawn, at the discretion of the Principal Investigator. Medical care including inpatient care if necessary will be offered.

- Acute disease at the time of vaccination. Acute disease is defined as the presence of a moderate or severe illness with or without fever. All vaccines can be administered to persons with a minor illness at the discretion of the investigators. Details of any minor illness will be recorded in the CRF.
- Axillary temperature of ≥37.5°C (99.5°F) at the time of vaccination.
- Receipt of routine EPI vaccination within one week of study vaccine

## 8. Study schedule and procedures

**Identification of Study Participants**

Community sensitisation will be undertaken to engage the community with the study and recruit volunteers for participation in the study. Volunteers will be assessed at screening visits to determine if they are eligible to participate in the study.

***Community sensitisation***

The CNRFP study team will hold local community meetings and explain the study to the parents/guardians of potentially eligible infants/children. During these meetings the investigators will explain the following: the need for a vaccine (including a simple picture of the burden of malaria on the community); the current status of vaccine development (including the fact that this is likely to be a prolonged process); the study screening and informed consent procedure; risks of vaccination and the unproven benefits of vaccination. It will be stressed that this is an experimental vaccine and cannot be guaranteed to provide protection, and that it will therefore still be necessary to seek treatment for possible malaria even after vaccination and continue to use other protective measures such as bed nets. It will be made clear that neither parents/guardians, nor investigators will know which vaccination regimen the child has received until the end of the study. It will be explained that a photograph of the infant/child and parent/guardian will be taken if they are eligible to be enrolled in the trial, to aid identification.

 © University of Oxford, 2013

After this meeting based on the list of infants/children of suitable age for participation in the trial drawn for the DSS database, parents/guardians will be asked to participate in a public lottery that is made to randomly select infants/children who will be invited for a screening visit.

A set of sealed envelopes containing "YES" or "NO" will be prepared. When the infant's/child's name is called by the investigator, the parent/guardian will come and randomly pick one envelope. If envelope containing a "YES" is picked, the infant's/child's name will be logged into the log sheet for screening. If it is "NO", the parent/guardian and infant/child will not be invited for screening. This method has been used in previous trials and is accepted by the communities as a fair way of giving the chance to each infant/child meeting the age criteria to be invited for the screening visit. All children thus selected will be invited to the Banfora trials center for the screening visit.

***Screening Visit***

We will provide detailed information about the study for distribution to the parents/guardians. The investigators will endeavour to ensure that all carers fully understand the risks. Any carer who appears to have less than complete understanding will be considered unable to give consent. As with any experimental vaccine the parents/guardians must understand that the vaccines have not yet been shown to prevent infection and this will be stressed during the recruitment stage. They must also understand the very small chance of anaphylactic reactions and thereby the importance of complying with the one-hour observation period after each vaccination. The information sheet covers these points in detail, and each parent/guardians will have the contents of the sheet explained in individual meetings.

If it is determined by the investigator conducting the screening visit that free and informed consent is given by the parent/guardian for their infant/child to participate in the trial, the parent/guardian will be asked to complete the consent form. The parent/guardian will thumbprint the consent form if illiterate.

A literate, impartial witness will be present for screening procedures and countersign the consent form if the parents/carers are illiterate.

Infants/children of parents/guardians who have consented will undergo the full screening procedures. This consists of medical history, physical examination, and blood sampling for screening tests as detailed below (Laboratory Evaluations).

The village of residence of the participant will be documented, along with the GPS coordinates of the homestead.

The investigator will determine whether the infant/child is eligible to participate in the study, using the findings at screening, including the results of the Screening blood tests. Infants/children eligible to participate in the study will fulfil all of the inclusion criteria, and meet none of the exclusion criteria.



 © University of Oxford, 2013

**Allocation of participants to Study Group and order of enrolment of Study Groups**

Participants are considered enrolled into the study when they have received the first study vaccination.

The study will commence with the Phase 1 lead-in Safety Evaluation. Thirty eligible participants will be identified as above, and allocated to Group A. The participants will be the following ages at enrolment:

- 5 to 8 months: 10 participants
- 9 to 12 months: 10 participants
- 13 to 17 months: 10 participants

For the Phase 2 Trial, 700 eligible participants will be identified as above, and randomised to Group B or C by an independent statistician, who is not one of the investigators. The randomisation will be stratified according to the following age categories, with varying block sizes:

- Participants aged 5-8 months at enrolment
- Participants aged 9-12 months at enrolment
- Participants aged 13-17 months at enrolment

A randomisation list will be generated and sequentially numbered opaque sealed envelopes will be made for the investigators to ensure treatment concealment. The investigators will not be aware of the randomisation result in the envelopes. The randomisation list, which will be password protected, will be kept in confidence until the final primary analysis is being conducted. As described in Section 7, all investigators will be blinded to allocation of participants in the Phase 2b trial to Group B vs Group C, except the staff responsible for the storage and preparation of the study vaccines.

The interim safety data to Day 21 of trial participation of Group A participants will be presented to the DSMB. DSMB review and approval will be required to administer the first vaccination to Group B and C participants.

The interim safety data to Day 63 of trial participation of Group A participants will be presented to the DSMB. DSMB review and approval will be required to administer the second vaccination to Group B and C participants.

**Study Visits**

Table 1 shows the window periods for the visits and outlines the study procedures at each visit for all study Groups.

**Day 0 (Vaccination)**

This visit will occur not more than 30 days following the screening visit. If more than 30 days have elapsed since screening, then a repeat Screening Visit will be conducted. Medical

 © University of Oxford, 2013

history, temperature monitoring +/- physical examination will be performed. Blood sampling will be performed as detailed below (Laboratory Evaluations).

Ongoing eligibility for participation will be confirmed according to the inclusion and exclusion criteria, prior to blood sampling and vaccination. Blood sampling will be performed as detailed below (Laboratory Evaluations).

Infants are considered enrolled into the study when they receive the first study vaccination. The vaccine will be administered as detailed in Section 7 and according to local SOPs.

Following vaccination, the vaccination site will be covered with a dressing which will be removed after 30 minutes. The volunteer will be monitored for one hour (or longer if necessary) after vaccination.

The CRF will be updated.

**Days 1, 2 and 3**

Each subject will be visited at home daily for three days by a field worker for assessment and recording of any solicited and unsolicited AEs. If necessary the volunteer will continue to be seen regularly until the AEs have resolved or stabilised.

**Day 7**

Medical history, temperature monitoring +/- physical examination will be performed. The CRF will be updated, including the records of AEs and concomitant medications.
If axillary temperature ≥ 37.5°C and/or History of fever within the last 24 hours, a blood smear will be obtained for malaria diagnosis.

**Day 21**

Medical history, temperature monitoring +/- physical examination will be performed. Blood sampling will be performed as detailed below (Laboratory Evaluations). The CRF will be updated, including the records of AEs and concomitant medications.

If axillary temperature ≥ 37.5°C and/or History of fever within the last 24 hours, a blood smear will be obtained for malaria diagnosis

**Day 56 (Vaccination)**

Medical history, temperature monitoring +/- physical examination will be performed. Ongoing eligibility will be confirmed by the Investigator according to the inclusion and exclusion criteria, prior to blood sampling and vaccination. Blood sampling will be performed as detailed below (Laboratory Evaluations).

Study vaccine will be administered as detailed in Section 7 and according to local SOPs. Following vaccination, the vaccination site will be covered with a dressing which will be

 © University of Oxford, 2013

removed after 30 minutes. The volunteer will be monitored for one hour (or longer if necessary) after vaccination.
The CRF will be updated, including the records of AEs and concomitant medications.

If axillary temperature ≥ 37.5°C and/or History of fever within the last 24 hours, a blood smear will be obtained for malaria diagnosis

**Days 57, 58 and 59**

Each subject will be visited at home daily for three days by a field worker for assessment and recording of any solicited and unsolicited AEs . If necessary the volunteer will continue to be seen regularly until the AEs have resolved or stabilised.

At the Day 57 visit, the fieldworker will document the bednet use and residual spraying.

**Day 63**

Medical history, temperature monitoring +/- physical examination will be performed.

Blood sampling will be performed as detailed below (Laboratory Evaluations). The CRF will be updated, including the records of AEs and concomitant medications.

If axillary temperature ≥ 37.5°C and/or History of fever within the last 24 hours, a blood smear will be obtained for malaria diagnosis

**Day 93**

Medical history, temperature monitoring +/- physical examination will be performed.
If axillary temperature ≥ 37.5°C and/or History of fever within the last 24 hours, a blood smear will be obtained for malaria diagnosis.

**Days 123, 153, 183, 213**

Each subject will be visited at home every 30 days by a field worker for assessment and recording of the subject health status. All the children found febrile/history of fever within the last 24 hours will be referred to the research centre where a blood smear will be obtained for malaria diagnosis. Any serious adverse event not detected will be documented. If necessary the volunteer will continue to be seen regularly until the AEs have resolved or stabilised.
At Study days 213 visit, the fieldworker will document the bednet use and residual spraying.

**Day 243**

Medical history, temperature monitoring +/- physical examination will be performed.

 © University of Oxford, 2013

Blood sampling will be performed as detailed below (Laboratory Evaluations). The CRF will be updated, including the records of SAEs and concomitant medications.

A blood smear will be obtained for malaria diagnosis

**Days 273, 303, 333, 363, 393**

Each subject will be visited at home monthly by a field worker for assessment and recording of the subject health status. All the children found febrile/history of fever within the last 24 hours will be referred to the research centre where a blood smear will be obtained for malaria diagnosis. Any serious adverse event not detected will be documented. If necessary the volunteer will continue to be seen regularly until the AEs have resolved or stabilised.
At Study days 393 visit, the fieldworker will document the bednet use and residual spraying

**Day 423**

Medical history, temperature monitoring +/- physical examination will be performed.

Blood sampling will be performed as detailed below (Laboratory Evaluations). The CRF will be updated, including the records of SAEs and concomitant medications.

A blood smear will be obtained for malaria diagnosis

**Days 453, 483, 513, 543, 573, 603, 633, 663, 693, 723, 753**

Each subject will be visited at home monthly by a field worker for assessment and recording of the subject health status. All the children found febrile/history of fever within the last 24 hours will be referred to the research centre where a blood smear will be obtained for malaria diagnosis. Any serious adverse event not detected will be documented. If necessary the volunteer will continue to be seen regularly until the AEs have resolved or stabilised.
At Study days 753 visit, the fieldworker will document the bednet use and residual spraying.

**Day 783**

Medical history, temperature monitoring +/- physical examination will be performed.

Blood sampling will be performed as detailed below (Laboratory Evaluations). The CRF will be updated, including the records of SAEs and concomitant medications.

A blood smear will be obtained for malaria diagnosis.

Information regarding the reported ethnolinguistic or tribal identity of the participant and their mother and father will be collected as part of the Add On Study 1 in one of the visits before the end of the follow-up period.. This information will be used specifically to aid interpretation of the genetic analyses.

 © University of Oxford, 2013

**Laboratory Evaluations**

Table 2, below, shows the Study Visits at which volunteers will have blood films for malaria diagnosis, and blood sampling for haematology, biochemistry, and exploratory immunology.


© University of Oxford, 2013

**Table 3: Timeline of Study Visits showing blood sampling and laboratory investigations for participants in Groups A, B, and C**

| | **SCHEDULE OF ATTENDANCES** | | | | | | | | | | | | | | |
|---|---|---|---|---|---|---|---|---|---|---|---|---|---|---|---|
| **Study Visit number** | **S** | **1** | **2-4** | **5** | **6** | **7** | **8-10** | **11** | **12** | **13-16** | **17** | **18-22** | **23** | **24-34** | **35** |
| **Clinic Visit** | X | X | | X | X | X | | X | X | | X | | X | | X |
| **Home visit** | | | X | | | | X | | | X | | X | | X | |
| **Day of Visit **** | D-30 to D-1 | D0 | D1, D2, D3 | D7 | D21 | D56 | D57, 58, 59 | D63 | D93 | D123, 153, 183, 213 | D243 | D273, 303, 333, 363, 393 | D423 | Days 453,483, 513,543, 573,603, 633,663, 693,723, 753 | D783 |
| **Window Period** | | | | -3/+7 | -6/+14 | ±14 | | -1/+7 | ±14 | ±14 | ±14 | ±14 | ±14 | ±14 | ±14 |
| **Blood Film for *P. falciparum*** | X | X | | X | | X | | X | | | X | | X | | X* |
| **Blood Film for *P. falciparum* if axillary temp ≥37.5 and/or history of fever within last 24 hours** | | X | X | X | X | X | X | | X | X | | X | | X | |
| **Blood sampling** | | | | | | | | | | | | | | | |
| *Group A volunteers* | | X | | | | X | | | | | | | | | |
| *Nested cohort of Group B and C volunteers* | | X | | | | | | | | | | | | | |
| *All volunteers* | X | | | | X | | | X | | | X | | X | | X |
| **Tests done on sampled blood** | | | | | | | | | | | | | | | |
| *Haematology and Biochemistry* | X | | | | X | | | X | | | X | | X | | X |
| *Exploratory Immunology* | | X | | | X$^{++}$ | X | | X$^{+}$ | | | X$^{+}$ | | X$^{+}$ | | X$^{+}$ |



 © University of Oxford, 2013

Each study visit will occur the indicated number of days from Day 0, within the window period for that visit S: Screening Visit; X: procedure takes place, D: Day.

* if efficacy analysis is extended to include data to 24 months following completion of vaccinations

$^{+}$: Group A volunteers, and nested cohort of Group B and C volunteers only

$^{++}$: Group A volunteers only

<u>Descriptions of Blood sampling and Laboratory Evaluations</u>

Blood films for *P. falciparum*: The blood film will be prepared with venous blood where possible, to minimise volunteer discomfort. Thick blood smears will be stained with Giemsa and read by experienced microscopists based on the CNRFP standard SOP.

Blood will be sampled at the visits indicated in Table 3, for haematology, biochemistry, and exploratory immunology. The volume of blood per blood sampling will be 5ml, except at each of the day 0,21 and 63 timepoints, where, if deemed safe by the investigators taking in to account any other blood tests done for the routine care of the infant and the infant's state of health, the investigators may collect 1ml/kg up to a maximum of 8ml.

l

Haematology: Full Blood Count. This will be done at the study visits as indicated in Table 3.

Buffy Coat: This will be fractionated from the whole blood sample at day 0 timepoint for all participants receiving the TRAP vaccine and 50 controls, and the remainder of control participants will have buffy coat fractionated from the blood sampled at day 423. This buffy coat will be used to extract DNA for the genetic analysis.

Biochemistry: including Creatinine, ALT and Bilirubin. This will be done at the study visits as indicated in Table 3.

Exploratory Immunology. This will be done at the study visits as indicated in Table 3. A nested cohort of Group B and C participants, who will have additional immunology compared to the other Group B and C participants, will be prepared by the statistician. This cohort will be a selection containing 340 Group B participants and 50 randomly selected Group C participants, so that the investigators remain blinded to Group B vs C allocation. The following investigations will be done on blood collected for exploratory immunology, at the discretion of the investigators:

- T cell enumeration and characterisation, using ELISPOT, and flow cytometry with intracellular cytokine staining
- Measurement of antibodies to TRAP and other malaria antigens, using ELISA
- Measurement of antivector immune responses
- Enumeration of antibody-secreting cells, using ELISPOT and flow cytometry
- Cytokine quantification in serum, using ELISA

 © University of Oxford, 2013

- Measurement of antibody responses to EPI vaccines administered to participants including diphtheria, pertussis, tetanus, polio and measles.
- Evaluation of genetic determinants of immune responses and vaccine efficacy using HLA typing, DNA and RNA analysis of polymorphisms and transcript levels, detection of haemoglobin gene variants, and other suitable methods
- Transcriptional profiling

Plasma and cells for exploratory immunology will be stored at -20°C and -192°C respectively.

**Provision of care to the study participants**

Study contact personnel will be available 24 hours a day at trial site clinic and at the different health facilities of the study population catchment areas, seven days a week, to attend consulting children. Children requiring inpatient care will be admitted to the hospital where study personnel will be posted. Laboratory and radiological investigation will be carried out when appropriate. If necessary, children requiring more specialized care (treatment or diagnostic procedures) will be transported to a referral hospital. Treatment for medical conditions will be given according to the standard treatment regimens locally. Any expenses including transport incurred by the parent(s)/guardian(s) of study participants for clinical care related to acute conditions will be borne by the trial according to the appropriate local arrangements. Long-term care for chronic conditions unrelated to study procedures will be delivered following local guidelines with no financial support from the trial.

**Malaria case management**

***Uncomplicated Malaria Cases***

Trial subjects with uncomplicated malaria will be treated according to SOPs and national guidelines.

***Severe Malaria Cases***

Trial subjects with severe malaria will be treated according to SOPs and national guidelines.

**Ascertainment of malaria Endpoints**

Collection of malaria endpoints for analysis of efficacy will begin at Day 63, which is 7 days following of completion of the vaccination regimen.

Clinically qualified investigators will adjudicate the presence of the endpoints of clinical *P. falciparum* malaria, severe *P. falciparum* malaria, and asymptomatic *P. falciparum* carriage, before they are unblinded to Group B vs C allocation.

<u>Case Detection for Clinical *P. falciparum* malaria</u>

 © University of Oxford, 2013

For the primary efficacy endpoint, passive case detection will be used and will consist of continuous availability of medical care at the trial site and at the community clinics to which trials participant villages belongs to.
All subjects presenting to health facilities in the study area will be evaluated as potential cases of clinical malaria disease. A blood sample for the evaluation of malaria parasites will be taken for all children who are reported to have had a fever within 24 hours of presentation or have a measured axillary temperature of ≥37.5°C. A *P. falciparum* rapid diagnosis test will be performed to guide immediate patient management. However, efficacy results will be based on blood slide reading. The research team will be available 24 hours/ day, 7 days a week. The participants' parents/guardians will be informed to bring the child to the health facility should the child be "unwell".

Case Detection for Severe *P. falciparum* malaria

A passive surveillance will be implemented. All subjects presenting for admission through the outpatient and emergency departments of hospitals in the study areas will be evaluated as potential cases of severe malaria disease. During the hospitalization, the subject's course will be monitored to capture the signs and biological parameters indicative of severe malaria disease. If the subject's condition changes from admission and he/she meets one of the criteria additional investigations, these will be performed.

**Safety follow-up**

Trained field workers under the supervision of the investigators will visit daily each enrolled child for days 1 to 3 post vaccination. If necessary the child will continue to be seen by the field worker on subsequent days for follow-up of adverse events. The field workers will visit the child at 30 day intervals as indicated on the Timeline of Study Visits (Table 1). In the event that the field worker finds any Grade 3 solicited general or unsolicited symptoms, the volunteer will be brought to the vaccination centre for examination by a study clinician. During the field worker visits, the children's parent(s)/guardian(s) will be asked retrospectively if any medical event that might be a SAE occurred since the last visit and this information will be recorded. Unreported SAEs detected in this way will be investigated and reported by the PI or delegate on the corresponding SAE.

If a study participant is reported to be unwell at the time of a visit, the field worker will advise the parents to report to the trial site clinic or the nearest health facility where a study nurse will be posted and will notify this referral to the clinical team for follow up. In the event that a study participant is seriously ill, the field worker will inform the PI or designate, and transport will be arranged, to the referral hospital (where a study physician is posted), if judged appropriate by the responsible clinician.

In case a study participant is unwell and referred to the trial site clinic or health facility, a duplicate blood film will be obtained should the volunteer present symptoms or signs compatible with malaria (axillary temperature ≥ 37.5°C, History of fever within the last 24 hours, loss of appetite, malaise, vomiting and diarrhoea etc).

 © University of Oxford, 2013

A study clinician will review the infant at the Clinic Visits, on Days 7, 21, 56, 63, 93, 243, 363, and 723, for full safety and reactogenicity assessment.

**Immunogenicity measurements**

These will consist of measurements of cellular responses using ELISPOT, flow cytometry and gene expression profiling and humoral responses using ELISA and a functional assay of antivector immunity.

The interferon-gamma (IFNγ) enzyme-linked immunospot assay (ELISPOT) can be performed in two ways; the *ex vivo* assay that enumerates effector memory T cells and which has correlated directly with protection in two mouse models of malaria[76]; and the cultured ELISPOT that measures central memory T cell responses and correlates with protection in the field trial of RTS,S/AS02 in the Gambia[77] and in sporozoite challenge studies of viral vector vaccinations in Oxford[29]. IFNγ secreted by T cells after interaction with infected liver cells has been shown to induce death of liver-stage parasites[17]. The *ex-vivo* assay will be used as the primary readout for vaccine immunogenicity in this study. PBMC will be stimulated with pools of 20mer peptides spanning the length of the ME-TRAP insert and overlapping by 10 amino acids. Additional information on T cell responses will be obtained by intracellular cytokine staining and flow cytometry to determine whether responding T cells are CD4+ or CD8+ and assess production of other cytokines such as IL-2 and TNFα or the degranulation marker CD107a.

RNA analysis may also be used to examine the profile of gene expression following vaccination and during exposure as there is mounting evidence that gene expression profiles can predict characteristics of the immune response to vaccination and may possibly be able to be used to prospectively determine vaccine efficacy[78].

Antibody responses to each of the other vaccines administered as part of EPI will be measured using established methods of serological IgG measurement. Examples of such techniques include ELISA and multiplexed Luminex immunofluorescent technologies which have been undertaken in numerous other studies in collaboration with institutions including the National Institute for Public Health and the Environment (RIVM) in the Netherlands and the Respiratory and Meningeal Pathogens Unit in Johannesburg, South Africa. Although there are no distinct guidelines in this field, If antibody levels are discovered to be deficient in any participant which may indicate the re-vaccination would be appropriate, efforts will be taken to revaccinate the child following independent confirmation. Such an initiative would be possible within the framework of the ongoing trial.

Immune responses to vaccination may be affected by genetic factors, therefore we may also assess sequence variation in DNA from vaccinees by sequencing or other genotyping methods. Some of these analyses may be undertaken at third party sites such as the Wellcome Trust Sanger Institute.

Any genetic analyses can result in a significant amount of data. Following the generation of such genetic data it is routine to deposit such data in international repositories such as the European Bioinformatics Institute (EBI) in Hinxton, UK. The data is stored securely and

 © University of Oxford, 2013

anonymously and stringent protocols are empowered to ensure the data is released only to researchers with a specific scientific question. We anticipate that the data will be used almost exclusively between the institutes involved in the data generation including Oxford, Cambridge, and Burkina Faso to investigate the response of the enrolled infants' to vaccination and their environment, considering infectious disease in particular. To facilitate an understanding of the genetic results in the context of the rarely studied Burkinabe population it is envisaged that the genetic data will be used in studies such as the African Genomes Variation Project and the Genome Diversity in Africa Project which are related international consortia developed to provide a catalogue and appreciation of the genetic heterogeneity of African populations.

**Data collection**

Adverse events will be documented in individual case report forms (CRFs) for each volunteer. They will be recorded under two headings; local and systemic. There will be documentation of concomitant medication, concomitant vaccination, non-serious adverse event documentation, serious adverse event documentation and study conclusion. Case report forms will be kept securely.

The following data will be collected for concomitant medications: medication name (generic name), dose, frequency and route; start and stop dates; and indication.
Concomitant medication will be recorded according to the time period below:

- Antimalarial drugs, Immune modifying drugs and blood transfusions will be captured for the duration of the trial.
- Antipyretics, analgesics, systemic antibiotics will be collected from dose 1 of vaccination until 1 month post dose 2.
- All vaccines administered, not specified in the study protocol, will be recorded for the duration of the trial.

**Study termination**

The study will be discontinued in the event of any of the following:

- New scientific information is published to indicate that volunteers in the study are being exposed to undue risks as a result of administration of the IMPs by any route of administration, or as a result of the follow-up schedule.
- Serious concerns about the safety of the IMPs arise as a result of one or more vaccine related SAE occurring in the subjects enrolled in this or any other ongoing study of the IMPs.
- For any other reason at the discretion of the Principal Investigator.

**Definition of the Start and End of the Trial**

The start of the trial is defined as the date of the first vaccination of the first volunteer. The end of the trial is the date of the last visit of the last volunteer.

 © University of Oxford, 2013

## 9. Assessment of scientific objectives

**Lead-in Safety Evaluation**
Assessment of the safety and reactogenicity of ChAd63 ME-TRAP / MVA ME-TRAP prime-boost vaccination will be undertaken by summary listing of all solicited and unsolicited local and systemic adverse events (including results of clinical laboratory investigations where deemed adverse events), considered possibly, probably, or definitely related to vaccination; and line listing of all SAEs.

**Phase 2b trial**

A full detailed statistical analysis plan will be developed prior to any unblinding of the data. The first data analysis will be performed after the last enrolled participant has reached 6 months after last vaccination. Analyses for the subsequent follow-ups will be carried out when all participants have reached at least 12 months and 24 months after the last vaccination, except extension of the efficacy analyses to 24 months will be dependent on the outcome of the efficacy analyses of the 12 month data. Results of these analyses will be disseminated accordingly.

Investigators performing the statistical analyses will be unblinded to Group B vs C allocation once all data to 6 months following enrolment of all volunteers is collected and data locked. Investigators not performing the analyses, for example those undertaking field work and interpreting adverse events and malaria endpoints, will remain blinded until the end of the study.

 © University of Oxford, 2013

The According-to-Protocol population for the statistical analyses are those participants who are eligible to participate, and received both allocated vaccines within the specified time window periods without any contraindications to vaccine administration.

***Primary analysis***

The primary analysis of efficacy will be comparison between Group B and C of the time to first episode of malaria meeting the primary case definition of clinical malaria episode over a period of 6 months of follow up after the last vaccination (ie, from Day 63 to 243). This will be done using survival analysis (e.g. Cox proportional hazard model), adjusting for any stratification factors (age, bednet use). Results will be presented as adjusted hazard ratio with appropriate 95% confidence intervals. Vaccine efficacy will be calculated as 1-HR. Kaplan-Meier methods will be used to visualize the time to first malaria event. The primary analysis will be based on the per-protocol population. An unadjusted analysis will also be carried out on the basis of intention-to-treat.

***Secondary analyses: Efficacy***

The following analyses of efficacy will be performed:

*-Protective efficacy against clinical malaria*

- Time to first episode of malaria meeting the primary case definition of clinical malaria episode over a period of 12, and 24* months of follow-up after the last vaccination.

- Proportion of participants with an episode of malaria meeting the primary case definition of clinical malaria episode, within the periods, 6, 12 and 24* months of follow-up after the last vaccination (ie, from study day 63 to 243, 63 to 423, and 63 to 738, respectively)

*-Efficacy against asymptomatic P. falciparum infection*

- Proportion of participants meeting the primary case definition of asymptomatic *P. falciparum* infection, at study days 243, 423 and 738*.

*-Efficacy against secondary case definitions of clinical malaria episode*

- Time to first episode of malaria meeting the secondary case definitions of clinical malaria episode over a period of 6, 12, and 24* months of follow-up after the last vaccination.

- Proportion of participants with an episode of malaria meeting the secondary case definitions of clinical malaria, within a period of 6, 12 and 24* months of follow up after the last vaccination

Time to first or only episode of clinical malaria meeting secondary endpoint definitions will be analysed as above.

 © University of Oxford, 2013

Variation in efficacy over time will be examined using Schoenfeld Residuals, and time dependent covariate analysis in Cox Regression.  If significant variation in efficacy over time is identified then efficacy will be presented as a summary figure over the full length of follow up, but also as a varying figure over time.

The proportion of children with asymptomatic parasitaemia at the cross-sectional surveys will be presented, and vaccine efficacy evaluated using logistic regression.

Multiple episodes of malaria will be examined using Poisson or negative binomial models on the ATP cohort (negative binomial if the data is over-dispersed) and adjusted incidence rate ratios calculated, adjusted by stratification factors. An unadjusted ITT analysis will also be conducted.

* Depending on the outcome of the efficacy analysis of the 12 month data, this may be extended to 24 months following completion of vaccination

***Safety analyses***

Assessment of the safety and reactogenicity of vaccinations will be undertaken by summary listing of all solicited and unsolicited local and systemic adverse events (including results of clinical laboratory investigations where deemed adverse events), considered possibly, probably, or definitely related to vaccination; and line listing of all SAEs.

Safety endpoints will be summarised for Group B and C. The number and percentage of patients in each group who have any local reaction will be compared (using the chi-squared test, and by calculating confidence intervals on differences in percentages). Similarly, the numbers who have any systemic reaction or SAE will be compared between groups. Analysis will also be carried out according to vaccine received.

***Immunogenicity Analyses***

Immunogenicity data will be analysed according to a detailed analytical plan.

***Exploratory Analysis***

The following analyses will be performed:

- Time to first episode of severe malaria meeting the primary and secondary case definitions of severe malaria over a period of 12 and 24* months of follow-up after the last vaccination.

- Proportion of participants with an episode of malaria meeting the primary and secondary case definitions of severe malaria,  within a period  of 12 and 24* months of follow up after the last vaccination

Analyses for efficacy against severe malaria will be similar to those described previously. These are exploratory analyses that are likely to have low statistical power.


© University of Oxford, 2013

## 10. Safety Reporting

Definitions

Definitions for the terms adverse event (or experience), adverse reaction, and unexpected adverse reaction have previously been agreed to by consensus of the more than 30 Collaborating Centres of the WHO International Drug Monitoring Centre (Uppsala, Sweden). Although those definitions can pertain to situations involving clinical investigations, some minor modifications are necessary, especially to accommodate the pre-approval, development environment.

The following definitions, with input from the WHO Collaborative Centre, have been agreed:

*Adverse Event*

Any untoward medical occurrence in a clinical investigation subject occurring in any phase of the clinical study whether or not considered related to the vaccine. This includes an exacerbation of pre-existing conditions or events, intercurrent illnesses, or vaccine or drug interaction. Anticipated day-to-day fluctuations of pre-existing conditions, including the disease under study, that do not represent a clinically significant exacerbation will not be considered adverse events. Discrete episodes of chronic conditions occurring during a study period will be reported as adverse events in order to assess changes in frequency or severity.

 © University of Oxford, 2013

Adverse events will be documented in terms of a medical diagnosis(es). When this is not possible, the adverse event will be documented in terms of signs and symptoms observed by the investigator at each study visit.

Pre-existing conditions or signs and/or symptoms (including any which are not recognised at study entry but are recognised during the study period) present in a subject prior to the start of the study will be recorded on the Medical History form within the subject's CRF.

*Adverse Drug Reaction (ADR)*

An ADR is any untoward or unintended response to a medicinal product. This means that a causal relationship between the study medication and an AE is at least a reasonable possibility, i.e., the relationship cannot be ruled out.

*Serious Adverse Event (SAE)*

A serious adverse event (experience) or reaction is any untoward medical occurrence that at any dose:

- results in death,
- is life-threatening,
  **Note:** The term "life-threatening" in the definition of "serious" refers to an event in which the patient was at risk of death at the time of the event; it does not refer to an event which hypothetically might have caused death if it were more severe.
- requires inpatient hospitalisation or prolongation of existing hospitalisation,
- results in persistent or significant disability/incapacity, or
- is a congenital anomaly/birth defect.

Medical and scientific judgment should be exercised in deciding whether expedited reporting is appropriate in other situations, such as important medical events that may not be immediately life-threatening or result in death or hospitalisation but may jeopardise the patient or may require intervention to prevent one of the other outcomes listed in the definition above. These should also usually be considered serious.

*Suspected Unexpected Serious Adverse Reactions (SUSARs)*

A SUSAR is a SAE that is unexpected and thought to be related to the investigational product. Administration of further vaccines within the trial will be suspended until a safety review is convened.

Collection of Adverse Events

At each visit all adverse events will be documented, including the following solicited local reactions at the injection site: swelling, redness/discoloration, and pain/limitation of limb movement.

The largest diameter through the injection site of any local redness/discoloration will be recorded in millimetres. The largest diameter through the injection site of local swelling will

 © University of Oxford, 2013

be recorded in millimetres. Severity of these local findings will be graded using the scales below:

Grading for swelling

| Grade | **Diameter [mm]** |
|---|---|
| 0 | 0 |
| 1 | < 20 |
| 2 | 20 – 50 |
| 3 | > 50 |

Grading for redness/discoloration

| Grade | **Diameter [mm]** |
|---|---|
| 0 | 0 |
| 1 | < 50 |
| 2 | 50 – 100 |
| 3 | > 100 |

If able to be ascertained in the infant, the presence and severity of local pain/limitation of limb movement at the site of vaccination will be determined using the following scale:

| **Grade** | **Description** |
|---|---|
| **0** | No pain at all |
| **1** | Painful on touch, no restriction in movement of limb |
| **2** | Painful when limb is moved |
| **3** | Unable to use limb due to pain |

Adverse events will only be recorded in the CRF if they occurred up to 30 days post vaccination, unless they meet the criteria for serious adverse event as outlined above. Serious adverse events will be collected throughout the study period.

Episodes of malaria detected as endpoints in the efficacy evaluation will not be reported as Adverse Events.

Follow-up of Adverse Events

All AEs will be followed until resolution of the signs or symptoms or laboratory changes occurs, or until a non-study related causality is assigned.

Subjects who have moderate or severe on-going adverse events at the completion of the study will be advised to consult a physician if the event is not considered to be related to the study vaccine. A follow-up visit will be arranged to manage the problem and to determine the severity and duration of the event, if it is considered to be related to the study vaccine. If appropriate, specialist review will be arranged by CNRFP investigators.

Any serious adverse event possibly related to the vaccine and occurring after trial termination should be reported by the investigator according to the procedure described below.

Reporting of Adverse Events

 © University of Oxford, 2013

Every SAE occurring throughout the trial must be reported by telephone, email or fax to the sponsor, LSM and DSMB by the investigator as soon as (s)he is alerted of it and within one working day, even if the investigator considers that the adverse event is not related to vaccination. The investigator will then complete a SAE report form as soon as possible and within five working days or seven calendar days.

Any relevant information concerning the adverse event that becomes available after the SAE report form has been sent (outcome, precise description of medical history, results of the investigation, copy of hospitalisation report, etc.) will be forwarded to the Sponsor in a timely manner. The anonymity of the subjects shall be respected when forwarding this information.

SAEs that are suspected to be related to the vaccine will be reported to the Ethics Committee within 15 calendar days of the site becoming aware of the event. If the event is fatal or life-threatening, the event will be reported within 7 calendar days.

Suspected unexpected serious adverse reactions (SUSARs) will be reported according to national regulatory guidelines. The sponsor pledges to inform the Authorities of any trial discontinuation and specify the reason for discontinuation.

The causal relationship between the AE and the product will be evaluated by the investigator. This interpretation will be based on the type of event, the relationship of the event to the time of vaccine administration, and the known biology of vaccine therapy. The following are guidelines for assessing the causal relationship:

No relationship:

No temporal relationship to study product; *and*

Alternate aetiology (clinical state, environmental or other interventions); *and*

Does not follow known pattern of response to study product

Unlikely relationship

Unlikely temporal relationship to study product; *and*

Alternate aetiology likely (clinical state, environmental or other interventions); *and*

Does not follow known typical or plausible pattern of response to study product

Possible relationship:

Reasonable temporal relationship to study product; *or*

Event not readily produced by clinical state, environmental or other interventions; *or*

Similar pattern of response to that seen with other vaccines

Probable relationship:

Reasonable temporal relationship to study product; *and*

Event not readily produced by clinical state, environment, or other interventions *or*

Known pattern of response seen with other vaccines

 © University of Oxford, 2013

Definite relationship:

Reasonable temporal relationship to study product; *and*

Event not readily produced by clinical state, environment, or other interventions; *and*

Known pattern of response seen with other vaccines

Grading the severity of adverse events

For adverse events other than local swelling, redness/discoloration and pain/limitation of limb movement, for which the severity scales are detailed above, AEs will be graded according to the DAIDS AE grading table, published in 2004[81]. The DAIDS AE grading table classifies adverse events into one of four grades, ranging from mild to potentially life-threatening. The DAIDS AE grading table has indications for each of over sixty clinical parameters and forty laboratory parameters for grading adult and paediatric AEs. The table also includes general guidelines for estimating the grade of parameters not explicitly listed. Each grade is described broadly below:

- Grade 1 (mild): awareness of a symptom, but the symptom is easily tolerated and causes no or minimal interference with usual activity.
- Grade 2 (moderate): discomfort enough to cause greater than minimal interference with usual activity.
- Grade 3 (severe): incapacitating; symptoms causing inability to perform usual activities; requires absenteeism or bed rest.
- Grade 4 (potentially life-threatening): symptoms causing inability to perform basic self-care functions OR medical or operative intervention is indicated to prevent permanent impairment, persistent disability or death.

The DAIDS AE grading scales for the solicited adverse events of fever and of loss of appetite (anorexia) are shown below,

Grading for fever

| Grade | Temperature (nonaxillary) |
|---|---|
| 1 | 37.7-38.6 $^{0}$C |
| 2 | 38.7-39.3 $^{0}$C |
| 3 | 39.4-40.5 $^{0}$C |
| 4 | >40.5 $^{0}$C |

Grading for loss of appetite

| Grade | Description |
|---|---|
| 1 | Loss of appetite without decreased oral intake |
| 2 | Loss of appetite associated with decreased oral intake without significant weight loss |
| 3 | Loss of appetite associated with significant weight loss |
| 4 | Life threatening consequences OR Aggressive intervention indicated [eg, tube feeding or total parenteral nutrition(TPN)] |

Laboratory tests will also be graded on the DAIDS AE grading table.

 © University of Oxford, 2013

## 11. Data Handling and Record Keeping

### Data Management

The Principal Investigator will be responsible for receiving, entering, cleaning, querying, analysing and storing all data that accrues from the study. Responsibility for this may be delegated to the data manager at CNRFP. The data will be entered into the subjects' CRFs. Data will be subsequently transferred to an electronic database for analysis.

If any changes to the protocol are necessary during the study a formal amendment will be presented to the sponsor prior to submission to the relevant ethical and regulatory agencies for approval unless to eliminate an immediate hazard(s) to study participant without prior ethics approval. Any unforeseen and unavoidable deviations from the protocol will be documented and filed in as a protocol deviation in the Trial Master File, with explanation.

### Data Capture Methods

Data capture will be on paper CRFs. The CRFs will be considered source documents as healthy volunteers will not have hospital case-notes.
Adverse events will be tabulated in an electronic database (OpenClinica®) for descriptive analysis.
Immunological data will be transferred to an electronic database for analysis without any volunteer identifier apart from the unique volunteer number.

### Types of Data

Data collected will include solicited and non-solicited adverse event data, concomitant medications, clinical laboratory and exploratory immunology data. Source documents will


© University of Oxford, 2013

include laboratory results and the case record file containing the case report forms for each volunteer as the healthy volunteers participating in this study may not have medical notes.

**Timing/Reports**

Annual Safety Report: Due on anniversary of Regulatory Approval – sent to Regulatory and Ethical Bodies
Annual Progress Report: Due on anniversary of Ethical Approval – sent to Ethics Committee

**Archiving**

The investigator must keep all trial documents for at least 15 years after the completion or discontinuation of the trial.

**Protocol Deviations**

Any unforeseen and unavoidable deviations from the protocol will be documented and filed in the study file with explanation.

 © University of Oxford, 2013

## 12. Data Access and Quality Assurance

**Direct Access to Source Data/Documents**

The principal investigator will provide direct access to the source data documents to the Ethics Committee, to the regulatory agency, and to authorised representatives of the sponsor, permitting trial-related monitoring and audits.

**Quality Assurance**

Modifications to the Protocol

Any amendments to the trial that appear necessary during the course of the trial must be discussed by the investigator and sponsor concurrently unless to eliminate an immediate hazard(s) to study participants. If agreement is reached concerning the need for a substantial amendment, it will be produced in writing by the sponsor and/or the investigator and will be made a formal part of the protocol. Any substantial amendment requires Ethics Committee approval, but non-substantial amendments do not.
All substantial amendments must also be communicated to Regulatory Authorities, if appropriate.
An administrative or non-substantial change to the protocol is one that modifies administrative and logistical aspects of a protocol but does not affect the subjects' safety, the objectives of the trial and its progress. An administrative change does not require Ethics Committee approval. However, the Ethics committee must be notified whenever an administrative or non-substantial change is made.
The investigator is responsible for ensuring that substantial amendments to an approved trial, during the period for which Ethics Committee approval has already been given, are not initiated without Ethics Committee review and approval except to eliminate apparent immediate hazards to the subject.

 © University of Oxford, 2013

**Monitoring**

Initiation Visit

An initiation visit will be performed before the inclusion of the first subject in the study. The Monitor will verify and document that the material to be used during the trial has been received and that the investigational team has been properly informed about the trial and regulatory requirements.

Follow-Up Visits

The Monitor will carry out regular follow-up visits. The investigator commits to being available for these visits and to allow the monitoring staff direct access to subject medical files, if existing, and CRFs. The Monitor is committed to professional secrecy.
During the visits, the Monitor may:

- Carry out a quality control of trial progress: in respect of protocol and operating guidelines, data collection, signature of consent forms, completion of documents, SAE, sample and product management, cold chain monitoring
- Inspect the CRFs, TMF and correspondent correction sheets

The Monitor will discuss any problem with the investigator and define with him the actions to be taken.

Close-out Visit

A close-out visit will be performed at the end of the trial. Its goals are to make sure that:

- The centre has all the documents necessary for archiving
- All unused material has been recovered
- All vaccines have been accounted for

 © University of Oxford, 2013

## 13. Ethical Considerations

### Ethical Review

Before the inclusion of the first participant in the study, the protocol must be approved by Ethical Review Committees in Burkina and Oxford (OXTREC).

### Informed Consent

Although consent from one parent is sufficient, mothers of potential participants will be encouraged to discuss the study with their husbands and to have his agreement before consent is obtained.

The written information is provided in French only and the field workers interpret the written information in a language the carers understand. The field workers involved in the informed consent discussion are trained on the study and the information sheet and consent form, and are trained to discuss the trial in the local languages the carers understand (Gouin, Karaboro, Dioula). The language of the consent process is documented on the consent form. If the carer is not able to read and write in French, an adult witness, impartial of the trial, will be present through the whole consent process and sign and date the consent form.

The infant or child's carer should give written/thumbprinted informed consent before the infant or child is included in the trial, after having been informed of the nature of the trial, the potential risks and their obligations. Informed consent forms will be provided in duplicate (original kept by the investigator, one copy kept by the subject's representative).

### Confidentiality

All blood results and adverse event data will be encoded in an electronic database and stored securely by the principal investigator.

### Inducement

There may be a perception amongst carers of infant/child subjects of benefit from physical examination, laboratory screening in the current study, in addition to free health care

 © University of Oxford, 2013

provided during the study period for non-vaccine related medical problems. We will also offer compensation for transport expenses for all study subjects.

We do not feel these benefits are excessive, and believe it would be unreasonable to request the cooperation of a population in regular employment or with childcare responsibilities without offering compensation for time.

## 14. Indemnity/Compensation/Insurance

**Indemnity**

Compensation for any injury caused by taking part in this study will be in accordance with the guidelines of the Association of the British Pharmaceutical Industry (ABPI). Broadly speaking the ABPI guidelines recommend that 'the sponsor', without legal commitment, should compensate participants without them having to prove that it is at fault. This applies in cases where it is likely that such injury results from giving any new drug or any other procedure carried out in accordance with the protocol for the study. 'The sponsor' will not compensate participants where such injury results from any procedure carried out which is not in accordance with the protocol for the study. Participants' right at law to claim compensation for injury where negligence can be proven is not affected. In this instance the University of Oxford is the Research Sponsor Institution.

**Compensation**

Carers of infants and children enrolled in the study will be offered compensation for transport expenses.

**Insurance**

Investigators participating in this trial will receive insurance coverage from the University clinical trials insurance policy.

 © University of Oxford, 2013

## 15. References

1. World Malaria Report 2009. Geneva, World Health Organisation, 2009. http://www.who.int/malaria/ world_malaria_report_2009/en/index.html
2. Sachs J, Malaney P. The economic and social burden of malaria. Nature 2002;415:680-5.
3. Das P, Horton R. Malaria elimination: worthy, challenging, and just possible. Lancet 2010; 376(9752): 1515-7.
4. White NJ. Antimalarial drug resistance. J Clin Invest 2004;113:1084-92.
5. Baird JK. Eliminating malaria- all of them. The Lancet, Early Online Publication, 29 October 2010. doi:10.1016/S0140-6736(10)61494-8
6. Bojang, K.A., et al., Efficacy of RTS,S/AS02 malaria vaccine against Plasmodium falciparum infection in semi-immune adult men in The Gambia: a randomised trial. Lancet, 2001. 358(9297): p. 1927-34.
7. Aponte, J.J., et al., Safety of the RTS,S/AS02D candidate malaria vaccine in infants living in a highly endemic area of Mozambique: a double blind randomised controlled phase I/IIb trial. Lancet, 2007. 370(9598): p. 1543-51.
8. Alonso, P.L., et al., Efficacy of the RTS,S/AS02A vaccine against Plasmodium falciparum infection and disease in young African children: randomised controlled trial. Lancet, 2004. 364(9443): p. 1411-20.
9. Bejon, P., et al., Efficacy of RTS,S/AS01E vaccine against malaria in children 5 to 17 months of age. N Engl J Med, 2008. 359(24): p. 2521-32.
10. Nussenzweig, R.S., et al., Protective immunity produced by the injection of x-irradiated sporozoites of plasmodium berghei. Nature, 1967. 216(5111): p. 160-2.
11. Hill, A.V., et al., Common west African HLA antigens are associated with protection from severe malaria. Nature, 1991. 352(6336): p. 595-600.
12. Hill, A.V., et al., Molecular analysis of the association of HLA-B53 and resistance to severe malaria. Nature, 1992. 360(6403): p. 434-9.
13. Todryk, S.M., et al., Correlation of memory T cell responses against TRAP with protection from clinical malaria, and CD4 CD25 high T cells with susceptibility in Kenyans. PLoS One, 2008. 3(4): p. e2027.
14. Khusmith, S., M. Sedegah, and S.L. Hoffman, Complete protection against Plasmodium yoelii by adoptive transfer of a CD8+ cytotoxic T-cell clone recognizing sporozoite surface protein 2. Infect Immun, 1994. 62(7): p. 2979-83.
15. Romero, P., et al., Cloned cytotoxic T cells recognize an epitope in the circumsporozoite protein and protect against malaria. Nature, 1989. 341(6240): p. 323-6.
16. Roestenberg, M., et al., Protection against a malaria challenge by sporozoite inoculation. N Engl J Med, 2009. 361(5): p. 468-77.

 © University of Oxford, 2013

17. Schofield, L., et al., Gamma interferon, CD8+ T cells and antibodies required for immunity to malaria sporozoites. Nature, 1987. 330(6149): p. 664-6.

18. Hoffman, S.L., et al., Sporozoite vaccine induces genetically restricted T cell elimination of malaria from hepatocytes. Science, 1989. 244(4908): p. 1078-81.

19. Weiss, W.R., et al., Cytotoxic T cells recognize a peptide from the circumsporozoite protein on malaria-infected hepatocytes. J Exp Med, 1990. 171(3): p. 763-73.

20. Bejon, P., et al., The induction and persistence of T cell IFN-gamma responses after vaccination or natural exposure is suppressed by Plasmodium falciparum. J Immunol, 2007. 179(6): p. 4193-201.

21. Hill AVS et al. Prime-boost vectored malaria vaccines: progress and prospects. Hum Vaccin, 2010. 6(1): 78-83.

22. Bejon P, Andrews L, Andersen RF, Dunachie S,Webster D, Walther M, et al. Calculation of liver-to blood inocula, parasite growth rates and preerythrocytic vaccine efficacy, from serial quantitative polymerase chain reaction studies of volunteers challenged with malaria sporozoites. J Infect Dis 2005; 191:619-26.

23. McConkey SJ, Reece WH, Moorthy VS, Webster D, Dunachie S, Butcher G, et al. Enhanced T-cell
immunogenicity of plasmid DNA vaccines boosted by recombinant modified vaccinia virus Ankara in
humans. Nat Med 2003; 9:729-35.

24. Dunachie SJ, Walther M, Epstein JE, Keating S, Berthoud T, Andrews L, et al. A DNA prime-modified
vaccinia virus ankara boost vaccine encoding thrombospondin-related adhesion protein but not circumsporozoite protein partially protects healthy malaria-naive adults against Plasmodium falciparum sporozoite challenge. Infect Immun 2006; 74:5933-42.

25. Webster, D.P., et al., Enhanced T cell-mediated protection against malaria in human challenges by using the recombinant poxviruses FP9 and modified vaccinia virus Ankara. Proc Natl Acad Sci U S A, 2005. 102(13): p. 4836-41.

26. Bejon, P., et al., Safety profile of the viral vectors of attenuated fowlpox strain FP9 and modified vaccinia virus Ankara recombinant for either of 2 preerythrocytic malaria antigens, ME-TRAP or the circumsporozoite protein, in children and adults in Kenya. Clin Infect Dis, 2006. 42(8): p. 1102-10.

27. Bejon, P., et al., Immunogenicity of the candidate malaria vaccines FP9 and modified vaccinia virus Ankara encoding the pre-erythrocytic antigen ME-TRAP in 1-6 year old children in a malaria endemic area. Vaccine, 2006

28. Bejon, P., et al., A Phase 2b Randomised Trial of the Candidate Malaria Vaccines FP9 ME-TRAP and MVA ME-TRAP among Children in Kenya. PLoS Clin Trials, 2006. 1(6): p. e29.

29. Keating SM, Bejon P, Berthoud T, Vuola JM, Todryk S, Webster DP, et al. Durable human memory T cells
quantifiable by cultured enzyme-linked immunospot assays are induced by heterologous prime boost immunization and correlate with protection against malaria. J Immunol 2005; 175:5675-80.

30. Moorthy VS, McConkey S, Roberts M, et al. Safety of DNA and modified vaccinia virus Ankara vaccines against liver-stage P. falciparum malaria in non-immune volunteers. Vaccine 2003;21:2004-11.

 © University of Oxford, 2013

31. Schneider J, Gilbert SC, Blanchard TJ, et al. Enhanced immunogenicity for CD8+ T cell induction and complete protective efficacy of malaria DNA vaccination by boosting with modified vaccinia virus Ankara. Nat Med 1998;4:397-402.
32. Reyes-Sandoval, A., et al., Single-dose immunogenicity and protective efficacy of simian adenoviral vectors against Plasmodium berghei. Eur J Immunol, 2008. 38(3): p. 732-41.
33. Sridhar, S., et al., Single-dose protection against Plasmodium berghei by a simian adenovirus vector using a human cytomegalovirus promoter containing intron A. J Virol, 2008. 82(8): p. 3822-33.
34. Eur J Immunol. 2008 Mar;38(3):732-41.Single-dose immunogenicity and protective efficacy of simian adenoviral vectors against Plasmodium berghei. Reyes-Sandoval A, Sridhar S, Berthoud T, Moore AC, Harty JT, Gilbert SC, Gao G, Ertl HC, Wilson JC, Hill AV.
35. Walther, M., et al., Safety, immunogenicity, and efficacy of prime-boost immunization with recombinant poxvirus FP9 and modified vaccinia virus Ankara encoding the full-length Plasmodium falciparum circumsporozoite protein. Infect Immun, 2006. 74(5): p. 2706-16
36. Reyes-Sandoval A et al. Prime-Boost Immunization with Adenoviral and Modified Vaccinia Virus Ankara Vectors Enhances the Durability and Polyfunctionality of Protective Malaria $CD8^+$ T-Cell Responses. Infect Immun, 2010. 78(1): 145-153.
37. Schneider, J., et al., A prime-boost immunisation regimen using DNA followed by recombinant modified vaccinia virus Ankara induces strong cellular immune responses against the Plasmodium falciparum TRAP antigen in chimpanzees. Vaccine, 2001. 19(32): p. 4595-602.
38. Mayr A, Stickl H, Muller HK, Danner K, Singer H. [The smallpox vaccination strain MVA: marker, genetic structure, experience gained with the parenteral vaccination and behavior in organisms with a debilitated defence mechanism (author's transl)]. Zentralbl Bakteriol [B] 1978;167:375-90.
39. Mahnel H, Mayr A. [Experiences with immunization against orthopox viruses of humans and animals using vaccine strain MVA]. Berl Munch Tierarztl Wochenschr 1994;107:253-6.
40. Draper SJ, Heeney JL. Viruses as vaccine vectors for infectious diseases and cancer. Nat Rev Microbiol;8:62-73.
41. Stickl H, Hochstein-Mintzel V, Mayr A, Huber HC, Schafer H, Holzner A. [MVA vaccination against smallpox: clinical tests with an attenuated live vaccinia virus strain (MVA) (author's transl)]. Dtsch Med Wochenschr 1974;99:2386-92
42. Akira S, Takeda K, Kaisho T. Toll-like receptors: critical proteins linking innate and acquired immunity. Nat Immunol 2001;2:675-80.
43. Walther M, Thompson FM, Dunachie S, et al. Safety, immunogenicity, and efficacy of prime-boost immunization with recombinant poxvirus FP9 and modified vaccinia virus Ankara encoding the full-length Plasmodium falciparum circumsporozoite protein. Infect Immun 2006;74:2706-16.
44. Bett AJ, Haddara W, Prevec L, Graham FL. An efficient and flexible system for construction of adenovirus vectors with insertions or deletions in early regions 1 and 3. Proc Natl Acad Sci U S A 1994;91:8802-6.
45. Top FH, Jr., Grossman RA, Bartelloni PJ, et al. Immunization with live types 7 and 4 adenovirus vaccines. I. Safety, infectivity, antigenicity, and potency of adenovirus type 7 vaccine in humans. J Infect Dis 1971;124:148-54.
46. Tatsis N, Ertl HC. Adenoviruses as vaccine vectors. Mol Ther 2004;10:616-29.
47. Kobinger GP, Feldmann H, Zhi Y, et al. Chimpanzee adenovirus vaccine protects against Zaire Ebola virus. Virology 2006;346:394-401.

 © University of Oxford, 2013

48. Casimiro DR, Chen L, Fu TM, et al. Comparative immunogenicity in rhesus monkeys of DNA plasmid, recombinant vaccinia virus, and replication-defective adenovirus vectors expressing a human immunodeficiency virus type 1 gag gene. J Virol 2003;77:6305-13.
49. Xiang Z, Gao G, Reyes-Sandoval A, et al. Novel, chimpanzee serotype 68-based adenoviral vaccine carrier for induction of antibodies to a transgene product. J Virol 2002;76:2667-75.
50. Catanzaro AT, Koup RA, Roederer M, et al. Phase 1 safety and immunogenicity evaluation of a multiclade HIV-1 candidate vaccine delivered by a replication-defective recombinant adenovirus vector. J Infect Dis 2006;194:1638-49.
51. Harro CD, Robertson MN, Lally MA, et al. Safety and immunogenicity of adenovirus-vectored near-consensus HIV type 1 clade B gag vaccines in healthy adults. AIDS Res Hum Retroviruses 2009;25:103-14.
52. Casimiro DR, Bett AJ, Fu TM, et al. Heterologous human immunodeficiency virus type 1 priming-boosting immunization strategies involving replication-defective adenovirus and poxvirus vaccine vectors. J Virol 2004;78:11434-8
53. Bruna-Romero O, Gonzalez-Aseguinolaza G, Hafalla JC, Tsuji M, Nussenzweig RS. Complete, long-lasting protection against malaria of mice primed and boosted with two distinct viral vectors expressing the same plasmodial antigen. Proc Natl Acad Sci U S A 2001;98:11491-6.
54. Grabenstein JD, Pittman PR, Greenwood JT, Engler RJ. Immunization to protect the US Armed Forces: heritage, current practice, and prospects. Epidemiol Rev 2006;28:3-26.
55. Farina SF, Gao GP, Xiang ZQ, et al. Replication-defective vector based on a chimpanzee adenovirus. J Virol 2001;75:11603-13.
56. Tatsis N, Tesema L, Robinson ER, et al. Chimpanzee-origin adenovirus vectors as vaccine carriers. Gene Ther 2006;13:421-9.
57. Tatsis N, Blejer A, Lasaro MO, et al. A CD46-binding Chimpanzee Adenovirus Vector as a Vaccine Carrier. Mol Ther 2007
58. Dudareva M, Andrews L, Gilbert SC, et al. Prevalence of serum neutralizing antibodies against chimpanzee adenovirus 63 and human adenovirus 5 in Kenyan Children, in the context of vaccine vector efficacy. Vaccine 2009;27:3501-4.
59. Pinto AR, Fitzgerald JC, Giles-Davis W, Gao GP, Wilson JM, Ertl HC. Induction of CD8+ T cells to an HIV-1 antigen through a prime boost regimen with heterologous E1-deleted adenoviral vaccine carriers. J Immunol 2003;171:6774-9.
60. Moorthy VS et al. Safety and immunogenicity of DNA/modified vaccinia ankara malaria vaccination in African adults. J Infect Dis 2003; 188:1239-1244
61. Clinical Study Report: Trial TB012: An open randomised dose selection study evaluating the safety, immunogenicity, and impact of a candidate TB vaccine, MVA85A, on the immunogenicity of the EPI vaccines administered simultaneously to healthy infants previously vaccinated with BCG. University of Oxford, 31 October 2010. Final study report submitted to Oxford Tropical Research Ethics Committee 23 November 2010.
62. Capone s et al. Immune responses against a liver-stage malaria antigen induced by simian adenoviral vector AdCh63 and MVA prime-boost immunisation in non-human primates. Vaccine. 2010 Oct 26 [Epub ahead of print]
63. Clinical Study Report: Trial TB014: A Phase II study evaluating the safety and immunogenicity of a new TB vaccine, MVA85A, in healthy children and infants after BCG vaccination at birth. University of Oxford, 1 March 2010. Final study report submitted to Oxford Tropical Research Ethics Committee 1 March 2010.

 © University of Oxford, 2013

66. Reyes-Sandoval A et al. Prime-Boost Immunization with Adenoviral and Modofied Vaccinia Virus Ankara Vectors Enhances the Durability and Polyfunctionality of Protective Malaria CD8+ T Cell Responses. Infect Immun 2010; 78(1): 145-153.
67. McConkey SJ et al. Enhanced T-cell immunogenicity of plasmid DNA vaccines boosted by recombinant modified vaccinia virus Ankara in humans. Nature Medicine. 2003; 9:729-735.
68. Webster DP et al. Enhanced T cell-mediated protection against malaria in human challenges by using the recombinant poxviruses FP9 and modified vaccinia virus Ankara. Proceedings of the National Academy of Sciences of the United States of America. 2005; 102(13): 4836-4841.
69. Olotu A et.al. Efficacy of RTS,S/AS01E malaria vaccine and exploratory analysis on anti-circumsporozoite antibody titres and protection in children aged 5–17 months in Kenya and Tanzania: a randomised controlled trial. Lancet Infect Dis 2011; 11: 102-09.
70. The RTS,S Clinical Trials Partnership. First Results of Phase 3 Trial of RTS,S/AS01 Malaria Vaccine in African Children. N Engl J Med 2011; 365: 1863-1875
71. Reyes-Sandoval A, Wyllie DH, Bauza K, Milicic A, Forbes EK, Rollier CS, Hill AVS. CD8+ T Effector Memory Cells Protect Against Liver-Stage Malaria. J Immunol 2011 (187) : 1347-1357.
72. Lalvani A, Aidoo M, Allsopp CE, Plebanski M, Whittle HC, Hill AV. An HLA-based approach to the design of a CTL-inducing vaccine against Plasmodium falciparum. Res Immunol 1994;145:461-8.
73. O'Hara GA et al. Clinical assessment of a recombinant simian adenovirus ChAd63: a potent new vaccine vector. J Infect Dis 2012 Mar; 205(5): 772-81
74. Plotkin SA, Wiktor T. Rabies Vaccination. *Ann Rev Med* 1978; 29:583-91
75. Cox JH, Schneider LG. Prophylactic Immunization of Humans Against Rabies by Intradermal Inoculation of Human Diploid Cell Culture Vaccine. Journal of Clinical Microbiology 1976 3(2): 96-101.
76. Plebanski, M., et al., Protection from Plasmodium berghei infection by priming and boosting T cells to a single class I-restricted epitope with recombinant carriers suitable for human use. Eur J Immunol, 1998. 28(12): p. 4345-55.
77. Reece, W.H., et al., A CD4(+) T-cell immune response to a conserved epitope in the circumsporozoite protein correlates with protection from natural Plasmodium falciparum infection and disease. Nat Med, 2004. 10(4): p. 406-10
78. Querec TD et al. Systems biology approach predicts immunogenicity of the yellow fever vaccine in humans, Nature Immunology. 2009; 10(1): 116-125.
79. Howie SRC. Blood sample volumes in child health research: review of safe limits. Bulletin of the World Health Organization. 2011 89(1): 46-53. Accessed 17 April 2012 at http://www.who.int/bulletin/volumes/89/1/10-080010-ab/en/index.html.
80. Ethical considerations for clinical trials on medicinal products conducted with the paediatric population: Recommendations of the Ad hoc group for the development of implementing guidelines for Directive 2001/20/EC relating to good clinical practice in the conduct of clinical trials on medicinal products for human use. Accessed 17 April 2012 at ftp://ftp.cordis.europa.eu/pub/fp7/docs/ethical-considerations-paediatrics_en.pdf.
81. Division of AIDS Table for Grading the Severity of Adult and Paediatric Adverse Events, Version 1.0, December 2004, (Clarification August 2009). [Internet] Accessed at http://rsc.tech-res.com/Document/safetyandpharmacovigilance/Table_for_Grading_Severity_of_Adult_Pediatric_Adverse_Events.pdf, 19 October 2012.

 © University of Oxford, 2013

 © University of Oxford, 2013
